# Ephemeropteran and Trichopteran Assemblages Vary Across a Subtropical Rainforest Altitudinal Gradient: Useful Indicators for Climate Change

**DOI:** 10.1002/ece3.73003

**Published:** 2026-02-02

**Authors:** D. Pagotto, C. Burwell, K. Turlington, F. Sheldon

**Affiliations:** ^1^ School of Environment and Science Griffith University Nathan Queensland Australia; ^2^ Queensland Museum Brisbane Queensland Australia; ^3^ Australian Rivers Institute Griffith University Nathan Queensland Australia

**Keywords:** altitude, caddisflies, climate change, headwater streams, mayflies

## Abstract

The subtropical rainforests of eastern Australia are expected to be greatly affected by climate change, with several studies predicting an upward shift in elevational distribution for many groups of fauna and flora. Freshwater streams have so far been neglected by most studies involving elevation, climate change and subtropical rainforest. This study is the first to explore changes in macroinvertebrates across an elevational gradient within subtropical streams to determine the effect of elevation. The study focussed on Ephemeroptera and Trichoptera (ET) and identified indicator taxa with the potential to be used for future monitoring of climate change. Stream macroinvertebrates, specifically of the Orders Ephemeroptera and Trichoptera, and environmental data was collected from pools, riffles and runs at 13 sites across three subtropical streams that spanned an elevation gradient from 300 m to 1100 m a.s.l. Water temperature, substrate composition, stream width and riparian canopy cover were the most notable environmental changes observed across the gradient. Trichopteran taxa richness was negatively correlated with elevation; however, ephemeropteran taxa richness did not respond to elevation. Water temperature, canopy cover, stream width and substrate composition explained the highest variation in ET assemblages across the gradient, with ET assemblages separating into distinct ‘low’ (300 m–500 m a.s.l.) and ‘high’ (700–900 m a.s.l.) assemblages; the 1100 m elevational zone was distinct, with an observed sharp decline in species richness. Elevation, along with reach scale environmental factors, are influential in structuring ET assemblages in subtropical rainforest streams, with specific ET taxa having the potential to be useful indicators of climate change in these systems.

## Graphical abstract

This figure provides the elevation distribution of Trichoptera and Ephemeroptera across the elevational gradient and clearly shows how specific morphospecies can be used as indicators.

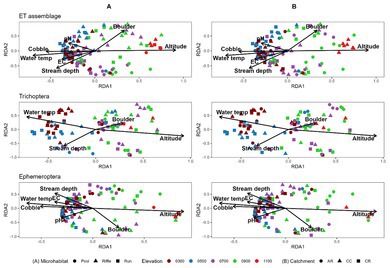

## 1 Introduction

Streams are longitudinally connected systems influenced by the unidirectional downstream movement of water (Ward 1989). This longitudinal form makes stream ecosystems unique; significant changes in elevation over short spatial distances in their headwater reaches generate predictable changes in microhabitat and physicochemical variables (Vannote et al. 1980; Frissell et al. 1986; Acosta and Prat 2010; Klinges and Scheffers 2021). These strong environmental and climatic shifts within short geographical distances (Nakamura et al. 2016) make elevational gradients useful tools for the study of ecosystem dynamics (Kitching et al. 2011). In headwater streams, both direct and indirect relationships between elevation and stream insect assemblage composition have been shown in temperate and tropical climate zones (Suren 1994; Jacobsen et al. 1997; Miserendino 2001; Henriques‐Oliveira and Nessimian 2010; Godoy et al. 2017); however, studies in subtropical climate zones are scarce (but see Jung et al. 2008; Jiang et al. 2010).

The inhabitants of headwater streams have usually evolved through isolation; they are often constrained to their resident stream by the hard and impenetrable boundary of the surrounding terrestrial landscape (Hughes et al. 2011) with many stream organisms having limited dispersal abilities across this boundary (Baggiano et al. 2011; Young et al. 2013). In response to strong longitudinal connection and weak lateral connection the assemblages of headwater streams often display high levels of endemicity (Baggiano et al. 2011; Young et al. 2013; Múrria et al. 2015; Hotaling et al. 2017). The biotic assemblages of streams at high elevation also show adaptations to the relatively cooler, more stable climatic conditions that headwaters provide (Hodkinson 2005). Numerous studies suggest it is the longitudinal change in stream water temperature along the elevational gradient that is the dominant driver influencing assemblage composition, with richness in insect order and family level taxonomy increasing linearly in response to increasing stream temperature and decreasing with increasing elevation (Jacobsen et al. 1997; Henriques‐Oliveira and Nessimian 2010). This makes headwater assemblages particularly vulnerable to climate change (Nukazawa et al. 2018).

Long‐term studies of European temperate streams show water temperature over the last few decades has increased on average by 1.88°C; and in response, warm‐ and moderately‐warm‐adapted aquatic insect assemblages increased in both abundance and richness while the abundance and richness of cold‐water adapted assemblages declined significantly (Haase et al. 2019; Baranov et al. 2020). Importantly, the thermal signature of the insect assemblages increased at a similar pace to the physical temperature of the stream, suggesting stream insects are linearly affected by increased warming (Haase et al. 2019). Similar patterns have been observed for mediterranean streams (Alba‐Tercedor et al. 2017), but there are few studies from the subtropics. This pattern underlies an ongoing process where cold‐water‐adapted species are replaced by warm‐water‐adapted species at a level of global warming of only 0.5°C, which is alarming considering the future temperature predictions of climate change. Most importantly, the strongest changes occurred along the headwater elevational gradient, suggesting stream invertebrates use the spatial arrangement of stream networks to colonise species‐specific novel temperature niches uphill, further threatening cooler‐water adapted communities (Haase et al. 2019).

The responses of stream insect assemblages to the effects of climate change are extremely complex and highly variable among orders, families, genera and even species (Chessman 2009; Li et al. 2013; Bush et al. 2014; Alba‐Tercedor et al. 2017; Pyne and Poff 2017; Frauendorf et al. 2019; Haase et al. 2019; Baranov et al. 2020). Increasing temperatures are expected to affect aquatic insects with low dispersal capacity such as mayflies (Ephemeroptera) and caddisflies (Trichoptera); however, there is evidence that even species with high dispersal abilities will be impacted through a reduction in suitable habitat. Dragonflies (Order Odonata) are known for their strong flight ability and thus high capacity for dispersal; however, a continental scale study of Australian Odonata under two future climate scenarios predicted a significant decrease in suitable habitat by 2085 for 56%–69% of all species (Bush et al. 2014).

The Gondwanan Rainforests of Australia's World Heritage Area comprise the largest tracts of subtropical rainforest in the world; however, their lowland remnants are highly fragmented. These rainforests have been described as ‘an archipelago of refugia’ for endemic flora and fauna. The ‘archipelago’ refers to the high number of restricted ecosystems present across an elevational gradient within these subtropical rainforests; if organisms are unable to move between these rainforest ‘islands’ they will be inherently vulnerable to climatic and environmental changes (Laurance et al. 2011). This region of Australia has the potential to be severely impacted by increasing temperatures associated with climate change, as well as an increase in the variability of rainfall (Laurance et al. 2011).

While the region is mountainous by Australian standards, the total elevation is restricted to below 1300 m above sea level (a.s.l.). This elevation restriction causes a ‘summit‐trap’ (Sauer et al. 2011), as temperatures increase cold water adapted species with limited dispersal ability literally run out of elevation in low‐elevation mountain ranges. This has been observed in Europe where 70%–80% of the distributional range of European freshwater montane communities are predicted to be lost by the end of the 21st century based on two IPCC climate scenarios (Sauer et al. 2011) with the same scenario likely in the subtropical rainforests of eastern Australia (Hagger et al. 2013).

Previous elevational studies in the Australian Gondwanan rainforest, specifically Lamington National Park, have focussed on terrestrial fauna (mostly arthropods) and plant groups (Ashton et al. 2011; Burwell and Nakamura 2011; Greenslade and Kitching 2011; Laidlaw et al. 2011; Ødegaard and Diserud 2011), with responses to elevation complex and variable. So, while the elevational response of terrestrial arthropods and vegetation has been investigated for this area, there have been no comparable studies on aquatic insect assemblages. This study aims to fill this gap and identify those assemblages and species likely to be impacted by climate change due to restricted elevational distributions. The study focusses specifically on mayflies (Ephemeroptera) and caddisflies (Trichoptera), two orders known to be sensitive to environmental change (Jacobus et al. 2019; Benhadji et al. 2025).

In this study, environmental changes within three stream catchments across an elevational gradient in the low‐range Lamington National Park, Australia, were quantified, including the effects of these changes on Ephemeroptera and Trichoptera assemblages. This was then used to identify significant indicator species of elevation throughout the park. Given the strong longitudinal nature of these streams at elevation, we predict that higher elevation assemblages will be distinct and more dissimilar to each other across catchments compared to lower elevation sites, with distinct indicator species for elevation zones. This suggests conservation implications for assemblages at higher elevation, even in these relatively lowland areas.

## 2 Methods

### 2.1 Study Area and Survey Sites

**FIGURE 1:**
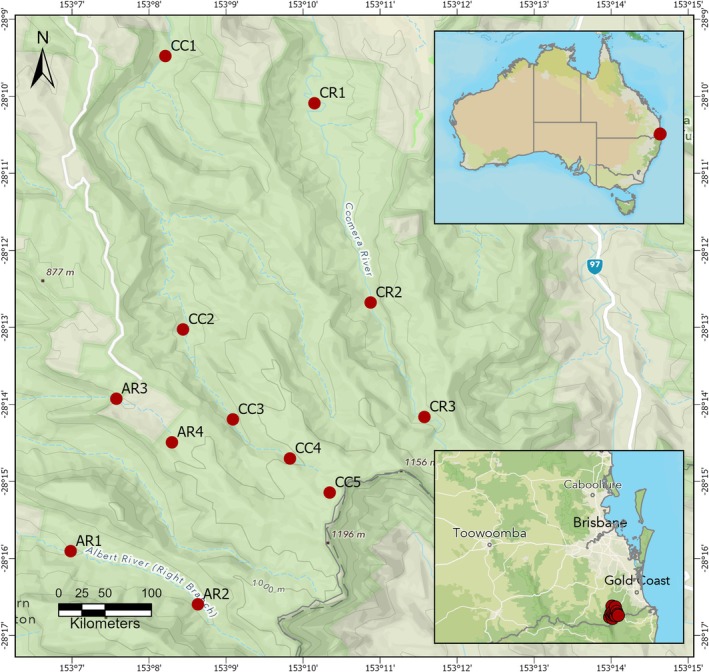
Map of Lamington National Park showing location of survey sites. The letter code identifies catchment whilst the number indicates which elevational zone the survey site represents. AR = Albert River; CC = Canungra Creek; CR = Coomera River. 1 = 300 m; 2 = 500 m; 3 = 700 m; 4 = 900 m; 5 = 1100 m.

This study was conducted in the three major catchments with headwaters in Lamington National Park (−28.16028°, 153.17973°; −28.34049°, 153.06417°), south‐east Queensland (SEQ), Australia (Figure 1). Lamington National Park is situated in the humid subtropics, classified as Cfa under the Koppen‐Geiger climate classification system (Kottek et al. 2006). Over the last 20 years, the park has had a mean annual rainfall of 1590 mm and a mean annual temperature range of 15.8°C–25.5°C (Binna Burra Alert station no. 40845 780 m a.s.l.: Bureau of Meteorology 2022). The park was created in 1915 to conserve a large area of mountainous forest along the Queensland‐New South Wales border and now protects over 23,000 ha of mostly broad‐leaved rainforest with patches of dry and wet sclerophyllous forest and heath. Lamington National Park was well suited for this study as it is uncommon to find such large areas of continuous subtropical rainforest along catchment gradients, much less three separate catchments in the one park.

**FIGURE 2:**
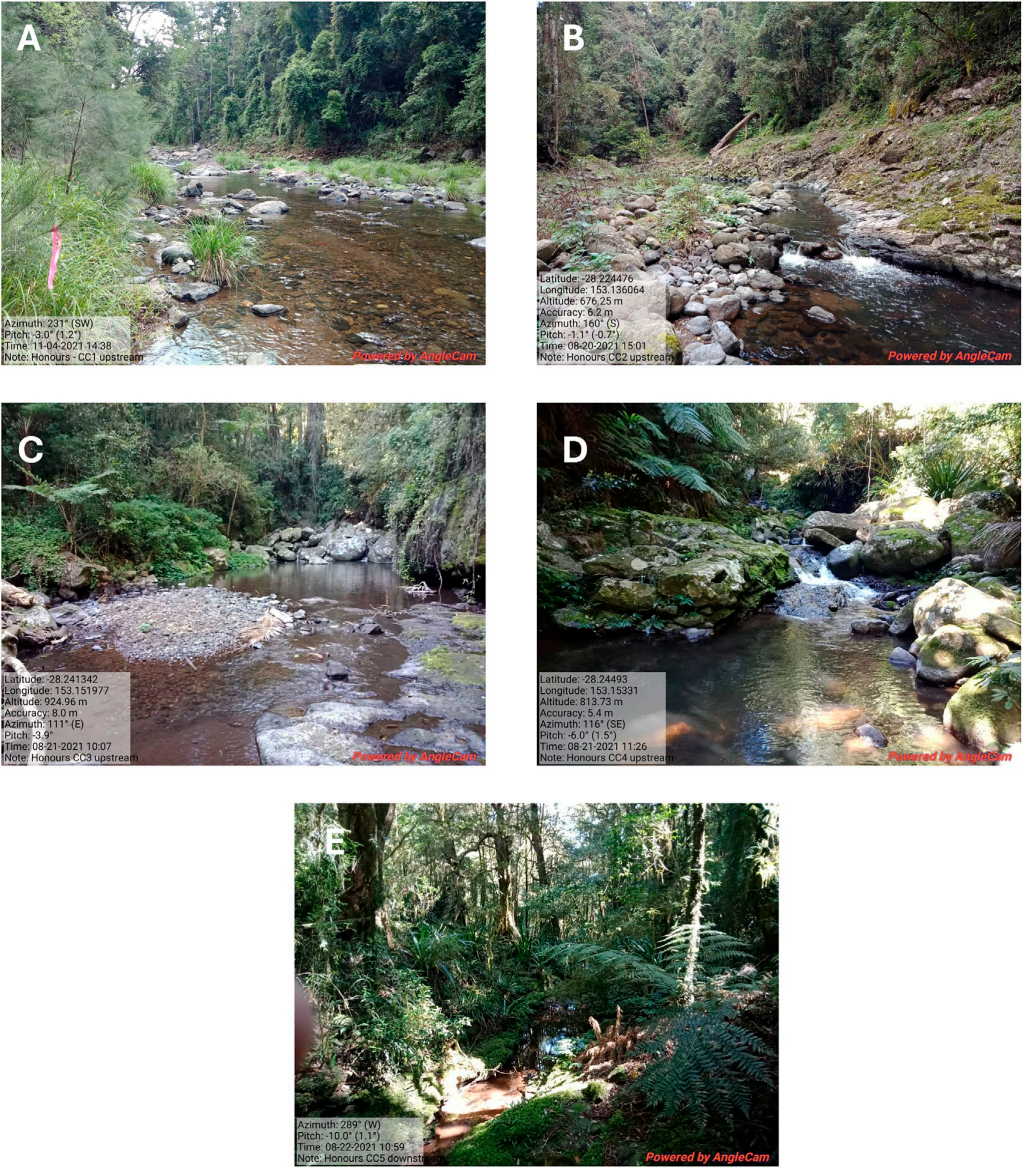
Representative site photographs across the elevational gradient. (A) Canungra Creek at 265 m (CC1); (B) Canungra Creek (right branch) at 495 m (CC2); (C) Canungra Creek (right branch) at 690 m (CC3); (D) Canungra Creek (right branch) at 850 m (CC4); (E) Toolona Creek at 1135 m (CC5).

Mostly permanent headwater streams within the Canungra Creek, Coomera River, and Albert River (AR) catchments formed the study area (Figure 2). All three catchments have an elevational range of at least 300–1000 m within the park; across this gradient, each waterway was divided into four or five elevational zones (sensu Kitching et al. 2011) that provided the survey sites for each catchment (300, 500, 700, 900, 1100 m a.s.l.). All waterways had similar channel characteristics and drained a comparable habitat gradient, with headwaters in temperate high‐elevation rainforest extending to lowland subtropical rainforest in lower reaches. Streams were small and narrow, with average stream depths ranging from 0.11 to 0.3 m and widths from 1.73 m at the high‐altitude site to 15.81 m at one low altitude site (Table 1). The most common regional ecosystems present across all three catchments were characterised by either *Lophostemon confertus* (R. Br.) open forest with a rainforest understory or variations on the complex notophyll vine forest vegetation community (regional ecosystem classification: 12.8.5/6/9; Queensland Government Regional Ecosystems Descriptions Database 2021).

**TABLE 1:** Site means for each in‐stream physicochemical and physical stream parameter. Site codes: AR = Albert River; CC = Canungra Creek; CR = Coomera River. 1 = 300 m; 2 = 500 m; 3 = 700 m; 4 = 900 m; 5 = 1100 m—m a.s.l. = metres above sea level.

Site	Altitude (m a.s.l.)	Water temperature (°C)	pH	Electrical conductivity (μS)	Stream depth (m)	Stream width (m)	Stream velocity (m/s)
AR1	350	20.85	5.87	84.6	0.30	7.50	0.09
AR2	440	18.84	5.73	98.5	0.15	8.86	0.14
AR3	775	16.14	5.65	130.1	0.23	5.03	0.09
AR4	885	15.42	5.53	92.5	0.15	5.20	0.16
CC1	265	20.88	5.63	106.1	0.18	15.81	0.33
CC2	495	16.52	5.83	65.6	0.22	13.80	0.41
CC3	690	14.93	5.82	61	0.21	8.00	0.25
CC4	850	14.14	5.83	60.5	0.19	4.70	0.49
CC5	1135	13.00	5.70	27.6	0.11	1.73	0.1
CR1	305	20.91	5.69	159.2	0.28	13.36	0.15
CR2	425	16.42	6.32	55.7	0.26	8.10	0.47
CR3	725	15.21	6.45	46.7	0.20	8.86	0.23
CR4	885	15.43	6.4	47.2	0.16	5.80	0.37

The study design considered the elevational zones as treatments and the three catchments as independent replicates (sensu Kitching et al. 2011) due to their geographical, geomorphological, and physiological similarities. In the CC catchment, five sites were sampled, one in each of the five elevational zones (300, 500, 700, 900 and 1100 m). The CR and AR catchments were both inaccessible above 1000 m; therefore, in these two catchments only four elevational zones were sampled (300, 500, 700 and 900 m).

Survey sites were identified using Queensland Globe (Queensland Globe 2022) based on topographical elevation, mapped habitat suitability and, where possible, site accessibility. The specific location of survey sites was confirmed through ground‐truthing where a 25 m reach was selected for each elevational zone across each of the three catchments giving a total of 13 survey sites (Appendix S1). The sites were identified based on criteria such as accessibility to the channel reach and presence of various microhabitats (i.e., riffles, pools and runs) and all were placed as close as possible to the elevational zone treatment they were aimed to represent.

Due to accessibility issues not all survey sites within a catchment were located on the same stream but were within the same sub‐catchment. The AR catchment was the most difficult to access at higher elevations, which resulted in the lower sites (300 and 500 m) being located on the right branch of the Albert River whilst the highest sites (700 and 900 m) were located on Morans Creek (Figure 1). The 1100 m site in the CC catchment was located on Toolona Creek as Canungra Creek was not accessible above 1000 m.

### 2.2 Sampling Design

Sampling was undertaken in the Austral spring and early summer (November to December 2021) across three separate sampling occasions, with each catchment sampled separately due to access. The region is often subject to high rainfall and river flows by mid‐ to late summer (Bureau of Meteorology 2021); therefore, this time of year provided an ideal balance between significant stream insect activity and the ability to sample safely for benthic insects. However, the east coast of Australia was subject to a La Niña event in the summer of 2021/2022, and the study sites subsequently experienced moderate‐high flows both during sampling (Bureau of Meteorology 2022).

Runs, riffles and pools are the primary geomorphic units in streams (Frissell et al. 1986) and were utilised as the three in‐stream microhabitat types for this study. Pools are relatively deep, with little to no flow; riffles are fast‐moving, shallow, highly oxygenated sections of the reach; and runs are relatively deep sections that contain moderate to fast‐moving laminar flows. The above descriptions were used to identify appropriate and consistent microhabitats to be sampled within each reach. Additionally, aquatic insects often display preferences for each of these microhabitats, and habitat selection is often associated with flow rate and levels of oxygenation (Meadows and Campbell 1972; Baptista et al. 2001; Mazzucco et al. 2015).

Each site was designated as a 25 m reach of stream. Ideally, within this reach three replicates of each microhabitat (run, riffle and pool) were identified and sampled individually totalling nine (9) potential in‐stream samples per survey site. However, this was not possible across all survey sites due to the natural variation in stream morphology and microhabitat occurrence. For example, at sites CR1 and CC5 there were only two suitable pool habitats within the reach and therefore only two pool samples were collected from these sites. A total of 113 samples were collected across the 13 survey sites (Appendix S1).

### 2.3 Data Collection

At each sampled microhabitat within each site across all three catchments the following water quality parameters were measured: water temperature (°C), pH, electrical conductivity (μS cm^−1^) and stream depth (m). Stream velocity (m s^−1^) was measured in the ‘run’ microhabitats. Stream width (m) was measured at the reach scale, replicated three times at 5, 12.5 and 20 m, then averaged. Water temperature, pH, and electrical conductivity were measured using a conductivity meter calibrated before sampling (TPS WP‐81 Meter) at a depth of 0.1–0.3 m depending on the depth of the microhabitat. Stream depth and width were measured using a ruler and tape measure respectively. The in‐stream substrate was classified as ‘boulder’, ‘cobble’, ‘gravel’, ‘sand’ or ‘silt’ and measured as estimated percent (%) cover within each 5 m^2^ area of microhabitat. In ‘runs’ water velocity was measured using the float method (Waterwatch Australia Steering Committee 2002). This method involves timing how long it takes for a tennis ball to float 1 m downstream using a metre ruler and a stopwatch, then applying the formula below, where velocity equals the distance travelled divided by the time taken.

$$\text{Velocity}=\frac{\text{Distance}}{\text{Time}}$$

Riparian habitat structure was assessed for each reach by measuring canopy cover (% cover over stream). Each measurement was taken in the middle of the stream using a convex spherical densiometer; for each site, measurements were replicated three times at 5, 12.5 and 20 m and then averaged.

Aquatic invertebrates were sampled via a combination of the kick‐net sampling technique and rock‐brushing; this is a semiquantitative approach. An area of approximately 5 m^2^ for each microhabitat was sampled via the kick‐net method for approximately 15 s using a standard 250‐μm D‐kick‐net (Sheldon et al. 2002). Kick‐netting involves the sampler disturbing the substrate with their feet, capturing suspended insects whilst working in a downstream–upstream direction to avoid contamination of subsequent samples. The contents of the net were then emptied into a collection container and marked appropriately according to the site, microhabitat code and sample number. Rock‐brushing was included in the sampling methodology to provide a more complete representation of stream insect occurrence and to capture those invertebrates difficult to dislodge from rocks with the kick‐net technique. A total of five similarly sized rocks (approximately 20cm^2^ in surface area each) were brushed lightly with a toothbrush into the collection container to remove any insects that were clinging to the rocks. Sample containers were filled with 70% aqueous ethanol and transported back to Griffith University and the Queensland Museum for further sorting and identification. For ease of sorting, samples were first filtered through nested sieves of various sizes (2000, 1000 and 250 μm) and identification focussed on each size increment. Many studies of river health use the richness of EPT taxa (Ephemeroptera, Plecoptera, Trichoptera) as a measure of ecosystem integrity (Sponseller et al. 2001; Park et al. 2003; Bunn et al. 2010). However, in this study very few Plecoptera (stoneflies) were found, so this study focusses on just Ephemeroptera and Trichoptera (ET) taxa. After initial sorting, the ET fauna were sorted into morphospecies, counted and identified. Identifications for ET fauna were made to the lowest taxonomic level possible using various dichotomous keys listed in Hawking (2000).

### 2.4 Data Analysis

All statistical analyses for this study were completed using the ‘vegan’ and ‘indicspecies’ packages in R (v.4.4.2). Due to the often‐high abundances of some stream insect taxa, all abundance data including all the taxa in this study were transformed to log_10_ (*x* + 1) (Sheldon et al. 2002; Marshall et al. 2006) unless otherwise specified.

To provide a measure of general community diversity, richness, and evenness across the elevational gradient, Shannon's diversity (SDI) and Pielou's evenness were calculated alongside adjusted species richness and abundance at the site level (Henriques‐Oliveira and Nessimian 2010). Species richness values were adjusted to 8 samples per site via rarefaction to account for those sites with a reduced number of microhabitat replicates. Two‐way analyses of variance (two‐way ANOVAs), assuming normal distributions, were used to assess if there was a significant effect of elevational zone (*p* < 0.05, df = 3, 11), catchment (*p* < 0.05, df = 2, 11), or an interaction between elevational zone and catchment (*p* < 0.05, df = 4, 11) on each of the above diversity measures at the scale of site. Site CC5 was excluded from these analyses as it was the only site in the 1100 m elevational zone.

**FIGURE 3:**
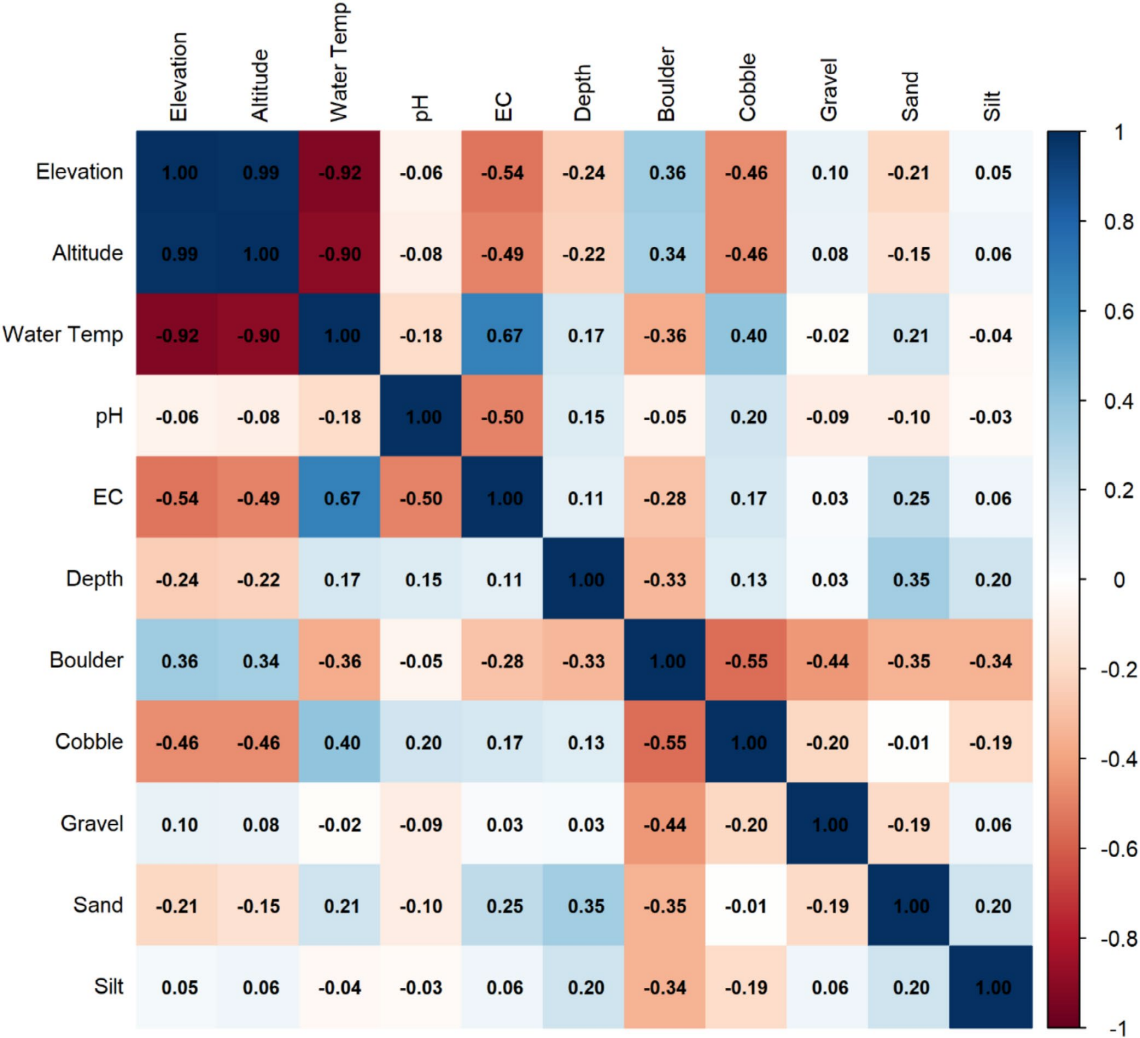
Pairwise Pearson correlation matrix of environmental variables used in the redundancy analysis (RDA). Correlation coefficients (*r*) are shown for all variable pairs, with colour intensity indicating the strength and direction of correlations. ‘Elevation’ refers to the ‘elevational zone’.

To examine how combinations of environmental variables jointly explain variation in macroinvertebrate community composition across microhabitats and catchments, redundancy analysis (RDA) was applied using the *vegan* package in R (v.4.4.2). The assemblage composition for the full trichopteran‐ephemeropteran assemblage and for each taxonomic subset representing the orders Trichoptera and Ephemeroptera was analysed. The assemblage composition data were transformed to use Euclidean distances in linear ordination and to reduce the influence of highly abundant taxa. Additionally, environmental variables were standardised (*z*‐score scaling) and screened for zero variance among sites and for strong collinearity using Pearson's correlation, with variable pairs flagged if they showed high correlation (|*r*| > 0.7; Figure 3).

As expected, elevational zone and altitude were highly correlated (*r* = 0.99), and both were strongly correlated with water temperature (*r* ≤ −0.9). Elevational zone was removed from the analysis, as altitude provides a continuous regional gradient that is directly linked to climatic and hydrological processes influencing stream temperature and flow regimes (Vannote et al. 1980). Altitude and water temperature were both retained due to their ecological relevance to macroinvertebrate assemblage structure, with altitude reflecting broad‐scale climatic gradients and water temperature representing local in‐stream conditions; effects were interpreted with caution given their shared variance.

The indicator value method (Dufrêne and Legendre 1997) was used to identify species that were characteristic (*p* < 0.05) of an elevational zone using the indicspecies package in R (*multipatt* function, 9999 permutations). This method draws from abundance and frequency of occurrence data to identify species characteristic of a particular treatment or site. The *IndVal* method in R provides an ‘A’ and ‘B’ component for each identified indicator species. Component ‘A’ or specificity is the likelihood or probability that the surveyed site belongs to a particular treatment given the fact that the species has been found there. A high specificity value of 1.0 would suggest that a species occurs within these treatments only. Component ‘B’ refers to the fidelity of the species to that treatment, or group of treatments. If a species has a fidelity value of 1.0, then it will occur in all sites belonging to that group. An indicator species should ideally be restricted to a particular elevational range (high specificity) and be readily detectable both spatially and temporally (high fidelity) (Nakamura et al. 2016).

## 3 Results

### 3.1 Environmental Characteristics

In‐stream physical and chemical parameters varied significantly across the elevational gradient and between catchments (Table 1). Water temperature ranged from 13°C at 1100 m (CC5) to as high as 20.91°C at 300 m at CR1. Stream pH showed less variability with a minimum of 5.53 at AR4 and a high of 6.45 at CR3. Electrical conductivity peaked at 159.2 μS cm^−1^ at 300 m at CR1 and dropped to as low as 27.6 μS cm^−1^ at 1100 m at CC5. Water temperature (*R* ^2^ = 0.84, *p* < 0.001), electrical conductivity (*R* ^2^ = 0.34, *p* < 0.05), stream depth (*R* ^2^ = 0.5, *p* < 0.01) and stream width (*R* ^2^ = 0.67, *p* < 0.001) were all significantly negatively related to elevation across the gradient. Stream velocity (*R* ^2^ < 0.0001, *p* > 0.05) and pH (*R* ^2^ = 0.007, p > 0.05) showed relatively minor variation and were not significantly related to elevation.

The stream substrate was dominated by boulders, and to a lesser extent by cobbles, both present at all sites across the gradient (Table 2). Sand was the least common substrate type and was more common at lower elevations, whilst silt was more randomly distributed throughout the gradient. Percent cover of cobbles and canopy were the variables most strongly related to altitude. Canopy cover showed a positive linear relationship (*R* ^2^ = 0.7, *p* < 0.001), while the presence of boulders and cobbles was positively (*R* ^2^ = 0.47; *p* < 0.01) and negatively (*R* ^2^ = 0.72, *p* < 0.001) related to altitude, respectively. The percent cover of sand (*R* ^2^ = 0.22, *p* > 0.05), gravel (*R* ^2^ = 0.01, *p* > 0.05) and silt (*R* ^2^ = 0.005, *p* > 0.05) were not significantly related to altitude.

**TABLE 2:** Mean percent composition of each substrate type and percent canopy cover for each site. Site codes: AR = Albert River; CC = Canungra Creek; CR = Coomera River. 1 = 300 m; 2 = 500 m; 3 = 700 m; 4 = 900 m; 5 = 1100 m—m a.s.l. = metres above sea level.

Site	Altitude (m a.s.l.)	Boulder	Cobble	Gravel	Sand	Silt	Canopy (% cover)
AR1	350	0.40	0.300	0.070	0.160	0.050	30.0
AR2	440	0.60	0.260	0.110	0.000	0.000	21.0
AR3	775	0.60	0.140	0.000	0.140	0.080	48.0
AR4	885	0.38	0.130	0.400	0.000	0.060	92.5
CC1	265	0.37	0.460	0.160	0.010	0.000	12.0
CC2	495	0.46	0.290	0.163	0.080	0.000	10.0
CC3	690	0.72	0.180	0.100	0.000	0.000	56.0
CC4	850	0.95	0.028	0.01	0.000	0.000	44.0
CC5	1135	0.85	0.075	0.075	0.000	0.000	83.0
CR1	305	0.32	0.400	0.150	0.085	0.000	8.0
CR2	425	0.53	0.370	0.040	0.000	0.040	19.0
CR3	725	0.47	0.375	0.100	0.000	0.025	70.0
CR4	885	0.58	0.100	0.240	0.000	0.025	54.3

### 3.2 Assemblage Composition

A total of 1722 individual Trichoptera larvae from 16 families and 45 morphospecies were collected across 113 samples from streams in Lamington National Park (Appendix S2). The three most abundant families collected across all three catchments were Glossosomatidae (27%), Hydropsychidae (24%) and Conoesucidae (16%). The most speciose families were Hydropsychidae (6 morphospecies), Hydrobiosidae (5 morphospecies) and Calocidae (5 morphospecies) with the families Antipodoeciidae, Calamoceratidae, Glossosomatidae, Helicopsychidae and Tasimiidae each represented by a single morphospecies. The glossosomatid *Agapetus* sp. AV1 was the single most abundant morphospecies, followed by *Diplectrona* sp. AV3 (Hydropsychidae), Genus Con B sp. AV2 (Conoesucidae) and *Tasimia* sp. (Tasimiidae).

A total of 3595 individual Ephemeroptera nymphs from 5 families and 29 morphospecies were collected across 113 samples (Appendix S2). The most abundant families collected were Leptophlebiidae (69%), Baetidae (24%) and Caenidae (6%). Leptophlebiidae were the most speciose (21 morphospecies); Baetidae and Caenidae each had three morphospecies, whilst Ameletopsidae and Vietnamellidae were represented by one morphospecies each. The leptophlebiid *Austrophlebiodes* sp. A was the single most abundant morphospecies, followed by other abundant taxa such as Baetid Genus 2 sp. (Baetidae) and *Ulmerophlebia* sp. AV2 (Leptophlebiidae).

#### 3.2.1 Relationships Between Diversity and Elevation

For Trichoptera, adjusted species richness significantly declined with increasing altitude (*R* ^2^ = 0.53, *p* < 0.01) as did Shannon's diversity (*R* ^2^ = 0.467, *p* = 0.01). Two‐way ANOVAs revealed significant differences between elevational zones for both adjusted species richness and Shannon's diversity; however, there was no significant difference in Pielou's evenness and abundance between elevation zones (Table 3). In contrast, two‐way ANOVA revealed no significant differences in adjusted species richness, Shannon's diversity, Pielou's evenness and abundance between catchments, indicating that catchment was not predictive of diversity measures. There was no significant interaction between elevational zone and catchment for any of the summary measures of diversity (Table 3).

**TABLE 3:** Trichoptera adjusted species richness, Shannon's diversity, Pielou's evenness and abundance at the site level; *n* = number of combined samples for each site. Two‐way ANOVAs were run between each diversity index and the elevational zone groupings (*p* < 0.05, df = 3, 11) as well as catchment (*p* < 0.05, df = 2, 11) and the interactions between the two groups (*p* < 0.05, df = 4, 11). The only significant interactions between elevational zone and diversity measures were for Pielou's evenness and abundance. No associations between catchment and diversity values were significant, and there was no significant interaction between the treatments.

Site	Catchment	Elevational zone	*n*	Adj. species richness	Shannon's diversity	Pielou's evenness	Abundance
AR1	Albert River	300	9	20	2.65	0.3106	86
CC1	Canungra Creek	300	9	16	2.43	0.3195	267
CR1	Coomera River	300	8	14	2.35	0.3459	79
AR2	Albert River	500	9	10	2.22	0.3749	60
CC2	Canungra Creek	500	8	18	2.26	0.3048	249
CR2	Coomera River	500	9	13	1.91	0.3465	246
AR3	Albert River	700	9	15	2.37	0.3186	118
CC3	Canungra Creek	700	9	16	2.57	0.3201	145
CR3	Coomera River	700	8	9	1.95	0.3849	64
AR4	Albert River	900	9	7	1.36	0.3231	102
CC4	Canungra Creek	900	8	12	2.03	0.3329	190
CR4	Coomera River	900	9	10	2.06	0.3562	95
CC5	Canungra Creek	1100	7	6	1.65	0.4369	21
Elevation: *F*‐value (*p*‐value)				**6.86 (0.03)**	**6.28 (0.03)**	0.28 (0.61)	0.29 (0.6)
Catchment: *F*‐Value (*p*‐value)				0.42 (0.42)	0.17 (0.68)	2.11 (0.18)	0.23 (0.64)
Elevation × catchment: *F*‐value (*p*‐value)				0.82 (0.39)	2.66 (0.14)	0.29 (0.6)	0.19 (0.67)

*Note:* CC5 was excluded from the two‐way ANOVA tests due to only having one replicate. Significant results are indicated by bold text.

In contrast, for Ephemeroptera, abundance showed a significant negative relationship with increasing altitude (*R* ^2^ = 0.33; *p* = 0.04) and was the only diversity measure to show any significant relationship. Two‐way ANOVA (Table 4) revealed no significant differences in diversity measures between elevational zones or catchments for adjusted species richness, Shannon's diversity, and abundance. Pielou's evenness was significantly different across elevational zones and catchments; however, there was no interaction between elevational zone and catchment for any of the diversity measures.

**TABLE 4:** Ephemeroptera adjusted species richness, Shannon's diversity, Pielou's evenness and abundance at the site level. *n* = number of combined samples for each site. Two‐way ANOVAs were run between each diversity index and the elevational zone groupings (*p* < 0.05, df = 3, 11) as well as catchment (*p* < 0.05, df = 2, 11) and the interactions between the two groups (*p* < 0.05, df = 4, 11). No interactions between elevational zone and diversity measures were significant except for Pielou's evenness. Only Pielou's evenness was significantly different between catchments; however, there were no significant interactions between elevational zone and catchment.

Site	Catchment	Elevational zone	*n*	Adj. species richness	Shannon's diversity	Pielou's evenness	Abundance
AR1	Albert River	300	9	10	1.85	0.32	233
CC1	Canungra Creek	300	9	11	2.00	0.32	615
CR1	Coomera River	300	8	14	1.88	0.29	479
AR2	Albert River	500	9	8	1.55	0.35	265
CC2	Canungra Creek	500	8	8	1.54	0.35	203
CR2	Coomera River	500	9	11	1.60	0.28	385
AR3	Albert River	700	9	9	1.84	0.35	417
CC3	Canungra Creek	700	9	9	1.85	0.34	228
CR3	Coomera River	700	8	11	1.90	0.34	141
AR4	Albert River	900	9	12	2.29	0.36	207
CC4	Canungra Creek	900	8	12	2.08	0.34	83
CR4	Coomera River	900	9	12	2.04	0.33	309
CC5	Canungra Creek	1100	7	2	0.68	0.71	30
Elevation: *F*‐value (*p*‐value)				0.04 (0.84)	2.95 (0.12)	**7.74 (0.02)**	4.58 (0.06)
Catchment: *F*‐Value (*p*‐value)				2.51 (0.15)	0.03 (0.86)	**9.28 (0.01)**	0.25 (0.63)
Elevation × catchment: *F*‐value (*p*‐value)				1.86 (0.2)	0.35 (0.56)	0.34 (0.57)	0.93 (0.36)

*Note:* CC5 was excluded from the two‐way ANOVA tests due to only having one replicate. Significant results are indicated by bold text.

#### 3.2.2 Elevational Patterns in ET Assemblage Composition

Altitude, water temperature, and stream depth consistently contributed to assemblage structure for the full Ephemeropteran‐Trichopteran (ET) assemblage, and for the Trichoptera and Ephemeroptera assemblages separately (Table 5). However, the amount of variation explained by the RDA differed among assemblages. The overall RDA explained 27% of the variation in the full ET assemblage (*n* = 7 significant environmental variables), 32% of variation in Ephemeroptera assemblages (*n* = 7), but only 21% of variation in Trichoptera assemblages (*n* = 3). Specifically, ephemeropterans were more sensitive to a wider range of environmental variables than trichopterans. Substrate effects differed among macroinvertebrate communities. Boulder and cobble substrate contributed to shaping the combined ET assemblage composition and ephemeropteran assemblage but showed weaker relationships with the trichopteran assemblage. Finer substrates (gravel, sand, and silt) did not significantly contribute to any assemblage composition.

**TABLE 5:** Permutation test results (999 permutations) for environmental variables included in redundancy analysis (RDA) models for the full ET, Trichoptera, and Ephemeroptera assemblages. For each assemblage, *F*‐statistics and *p*‐values are shown for each environmental variable. The model *r* ^2^ values represent the proportion of total variation explained by each RDA model.

Variable	ET assemblage		Trichoptera only		Ephemeroptera only	
	*F*	*p*	*F*	*p*	*F*	*p*
Altitude	15.7640	0.001***	11.0066	0.001***	20.9033	0.001***
Water temp	3.8432	0.001***	3.9115	0.001***	3.8583	0.001***
pH	2.1484	0.021*	1.6159	0.086	2.8633	0.017*
EC	3.1084	0.002**	0.7690	0.706	3.8805	0.001***
Stream depth	4.7015	0.001***	3.8459	0.001***	4.8711	0.001***
Boulder	3.1114	0.004**	1.7107	0.065	4.6872	0.003**
Cobble	1.9608	0.041*	1.4126	0.152	2.6293	0.012*
Gravel	1.1316	0.301	0.9931	0.448	1.3153	0.220
Sand	0.6135	0.852	0.5600	0.904	0.5849	0.767
Silt	1.4710	0.135	1.0831	0.364	1.8136	0.106
*r* ^2^	0.2746		0.2120		0.3216	

*Note:* Significance codes indicate the *p*‐value thresholds, where ***p < 0.001, **p < 0.01, *p < 0.05, and *p* < 0.01.

**FIGURE 4:**
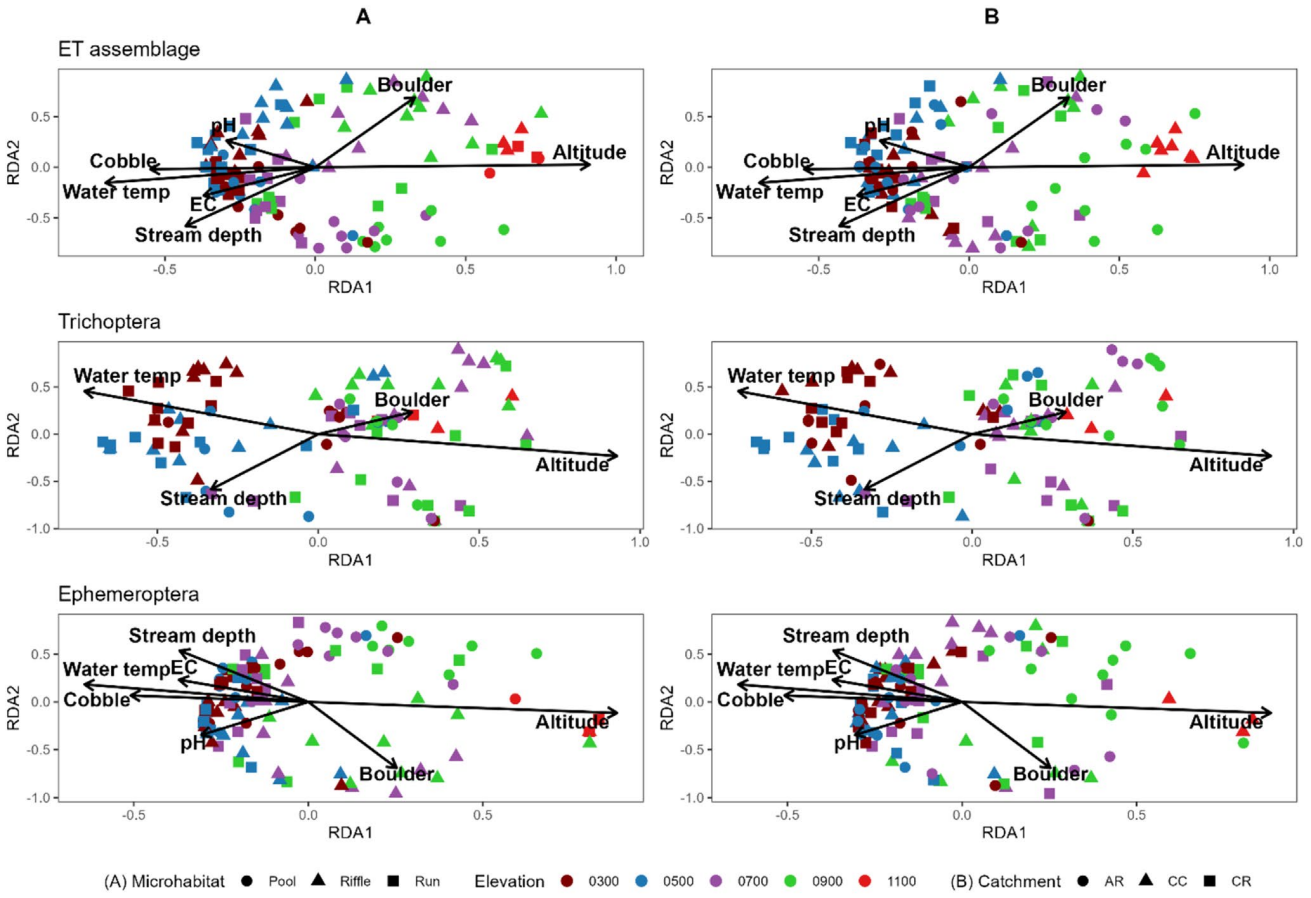
Redundancy analysis (RDA) ordination plots showing relationships between assemblage composition and environmental variables in Lamington National Park for the full ET assemblage, Trichoptera, and Ephemeroptera assemblage separately. Points represent sampling sites and are coloured by elevation category and shaped by either microhabitat in column A (left) and catchment in column B (right). Arrows indicate environmental variables retained by permutation testing (*p* < 0.05), with arrow direction and length reflecting correlations with the constrained ordination axes.

RDA1 and RDA2 explained 13.5% and 5.6% of variance in the full ET assemblage, 9.7% and 4.2% in the Trichoptera assemblage, and 17.8% and 7.3% in the Ephemeroptera assemblage, respectively (Figure 4). Across all ordinations, altitude aligned with positive RDA1, whereas water temperature aligned with negative RDA1. In the full ET assemblage, electrical conductivity, depth, and percent cobble substrate also aligned with negative RDA1, while boulder substrate and pH aligned more strongly with RDA2. In the Trichoptera assemblage, depth aligned primarily with negative RDA2, with fewer environmental vectors retained overall. In contrast, the ephemeropteran assemblages showed greater structuring along both axes, with electrical conductivity and percent cobble aligning with negative RDA1, and depth, percent boulder substrate, and pH showing opposing loadings along RDA2.

There was a clear separation of sites by elevational zone in the full ET and Ephemeroptera assemblage (Figure 4). Sites at elevations > 1100 m clustered toward positive RDA1, whereas lower elevational zones (< 700 m) clustered toward negative RDA1. Sites in the 900 m elevation zone were more broadly distributed across both axes for all assemblages. In the Trichoptera ordination, lower elevational zones (< 500 m) showed greater separation from other elevation zones, with sites in the 300 m zone aligning with higher RDA2 values and sites in the 500 m zone aligning with lower RDA2 values.

Environmental vector lengths differed among assemblages. Vectors for altitude, boulder substrate, depth, and water temperature were longer than those for pH, cobble substrate, and electrical conductivity in the full ET and Ephemeroptera assemblages, whereas vector lengths were more similar in the Trichoptera ordination. Patterns observed in the ordinations were consistent with our permutation test results (Table 5).

### 3.3 Indicator Species Across Elevation Zones

**FIGURE 5:**
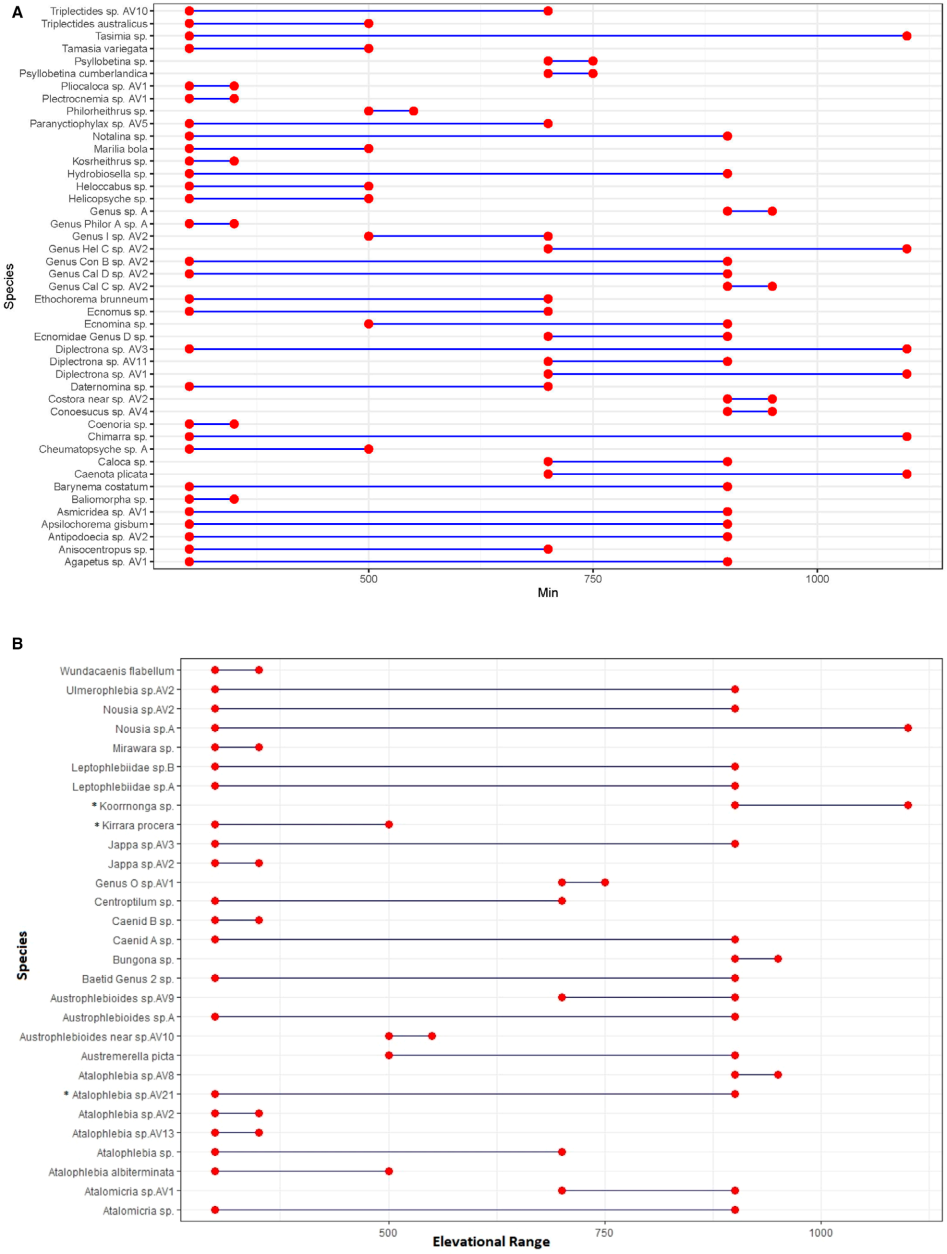
Range plot displaying the elevational distribution of (A) Trichoptera and (B) Ephemeroptera morphospecies. Morphospecies with an asterisk (*) next to them were identified as indicator species for elevational zones through the indicator value method (see also Table 6).

For Trichoptera the indicator value method identified five morphospecies associated to a particular elevational zone or group of zones across the gradient (Table 6, Figure 5A). The morphospecies identified were *Anisocentropus* sp. and *Apsilochorema gisbum* Mosely (300 and 700 m zones), *Helocabbus* sp. (300 m), *Cheumatopsyche* sp. (300 and 500 m zones) and *Diplectrona* sp. AV3 which was an indicator of elevational zones 500 m and higher. All indicator species identified had high fidelity, suggesting they are likely to occur at every site within their associated elevational zone (Table 6). *Cheumatopsyche* sp. had both high specificity and fidelity to the 300–500 m zone suggesting it is a morphospecies that only occurs within this zone of the elevational gradient based on our sampling methodology.

**TABLE 6:** Indicator species based on elevational range with a significance level of < 0.05. Morphospecies are ordered alphabetically by Family for Trichoptera and Ephemeroptera. Specificity ‘A’, is the likelihood or probability that the surveyed site belongs to a particular elevation zone given that the species has been found there. A specificity value of 1.0 would suggest that a species occurs within these elevation zones only. Fidelity ‘B’, refers to the fidelity of the species to that particular elevation zone, or group of elevation zones. If a species has a fidelity value of 1.0, then it will occur in all sites within that elevation zone.

	Species	Specificity (A)	Fidelity (B)	Significance	Elevational zone (s)
Trichoptera					
Calamoceratidae	*Anisocentropus* sp.	0.9521	1.0	0.012	300 m + 700 m
Helicophidae	*Helocabbus* sp.	0.8364	1.0	0.024	300 m
Hydrobiosidae	*Apsilochorema gisbum*	0.9194	1.0	0.013	300 m + 700 m
Hydropsychidae	*Cheumatopsyche* sp.	1.0	1.0	0.0025	300 m + 500 m
	*Diplectrona* sp. AV3	0.9805	1.0	0.015	500 m + 700 m + 900 m + 1100 m
Ephemeroptera					
Leptophlebiidae	*Atalophlebia* sp. AV21	0.96	1.0	0.006	700 + 900 m
	*Kirrara procera*	1.0	1.0	0.02	300 + 500 m
	*Koorrnonga* sp.	1.0	1.0	0.004	900 + 1100 m

For Ephemeroptera the indicator value method identified three morphospecies of Ephemeroptera associated to a particular group of elevational zones across the gradient (Table 6). The morphospecies identified as indicators were *Atalophlebia* sp. AV21 (700 and 900 m zones), *Kirrara procera* (300 and 500 m zones) and *Koornoonga* sp. (900 and 1100 m zones). All species identified had high fidelity, suggesting they are likely to occur at every site within their associated elevational range (Table 5). *Kirrara procera* Harker and *Koorrnonga* sp. had a high specificity suggesting that these morphospecies only occur within the 300–500 m and 900–1100 m zone of the elevational gradient, respectively (Table 6, Figure 5B).

## 4 Discussion

### 4.1 Characteristics of the Elevational Gradient

This is the first study to describe the elevational stratification of aquatic insect communities in Australia and specifically in subtropical rainforests. Other studies have focussed on stream invertebrates in different climatic zones (Jung et al. 2008; Jiang et al. 2010) or at extreme high elevations such as the Peruvian Andes (2500 m–4400 m a.s.l.) (Acosta and Prat 2010). In Australian subtropical rainforests, the focus has been on terrestrial arthropods and plants (Kitching et al. 2011).

The environmental changes observed in this study reflect those expected across a typical elevational gradient in nearly all stream systems: water temperature decreased with increasing elevation, canopy cover increased with elevation and stream width, and stream depth decreased with increasing elevation (Dodds et al. 2015, 2019). The observed negative relationship between decreasing water temperature and increasing elevation has been consistently found in studies of elevational gradients in well forested stream systems (Jacobsen et al. 1997; Jacobsen 2003; Henriques‐Oliveira and Nessimian 2010). However, the observed change in electrical conductivity across the elevational gradient should be treated with caution and is likely more closely related to catchment specific geological properties and local land use (Schmidt et al. 2012; Yegon et al. 2021). The increased proportion of cobble and boulders at higher elevations is consistent with microhabitat structuring in headwater streams (Frissell et al. 1986).

### 4.2 Assemblage Changes Across the Elevational Gradient

Summary measures of diversity for the Ephemeroptera and Trichoptera assemblages showed differing responses to elevation, with ephemeropteran abundance negatively associated and no abundance patterns observed for trichopterans. A negative relationship between aquatic insect abundance and elevation has been observed elsewhere (Jung et al. 2008; Castro et al. 2019), however, it is unclear how elevation is influencing abundance. For trichopterans, species richness and Shannon's diversity showed a negative relationship with elevation; however, there were no significant elevation‐related relationships in ephemeropteran species richness and Shannon's diversity. Interestingly, both negative (Suren 1994; Jacobsen et al. 1997, 2003; Jacobsen 2003, 2007; Castro et al. 2019) and positive (Jiang et al. 2010) relationships between aquatic macroinvertebrate assemblage diversity across elevational gradients, inclusive of Trichoptera and Ephemeroptera, have been observed elsewhere.

Assemblage composition of both the Ephemeroptera and Trichoptera was influenced by both elevation and microhabitat preference; however, elevation appeared to be the primary driver, explaining most variation in assemblage composition across the gradient. This is consistent with elevational studies of invertebrate assemblages in both terrestrial (Burwell and Nakamura 2011; Greenslade and Kitching 2011; Lambkin et al. 2011) and other aquatic systems (Suren 1994; Jacobsen et al. 1997; Henriques‐Oliveira and Nessimian 2010) elsewhere. Generally, our results suggested that the ET assemblage could be characterised into ‘low’ elevation (300–500 m) and ‘high’ elevation (700–900 m) groups. The 1100 m site formed a distinct assemblage which most likely reflected a lack of replicates within this elevational zone and the observed sharp decline in species richness, as opposed to the exclusive presence of species.

There was a strong relationship between elevation and water temperature but also strong relationships with electrical conductivity and aspects of substrate composition. Therefore, elevation‐related changes in assemblage composition are likely not influenced by any one variable. Water temperature has been identified by others as a primary driver in structuring stream macroinvertebrate assemblages along elevational gradients across a broad range of sites (Ward and Stanford 1982; Henriques‐Oliveira and Nessimian 2010; Haase et al. 2019; Baranov et al. 2020), with our study supporting this. Likewise, electrical conductivity, stream velocity and substrate composition have been identified as drivers of assemblage change across elevation gradients (Md Rawi et al. 2014; Godoy et al. 2017; Nguyen et al. 2018; Principe et al. 2019; dos Reis Oliveira et al. 2020).

Precipitation, and consequently water velocity, is known to play an important role in assemblage composition of streams in areas with variable rainfall, such as those with defined ‘wet’ and ‘dry’ seasons (Jacobsen and Encalada 1998; Monk et al. 2006) which include the streams in Lamington National Park and the subtropical rainforests of eastern Australia (Strong et al. 2011). The survey sites in this study were subject to relatively high flows during the sampling period caused by increased rainfall associated with the La Niña summer wet season in eastern Australia, which may have influenced observed abundance patterns; however, repeated sampling across different wet and dry periods would be needed to understand long‐term climate cycles on the elevational changes. Previous studies on the elevational distribution of terrestrial taxa conducted through the IBISCA Queensland project in Lamington National Park found different relationships between elevation and community structure depending on taxa, with some responses strongly influenced by seasonality and life history traits (Burwell and Nakamura 2011; Lambkin et al. 2011; Boulter et al. 2011). Longer sampling periods covering multiple seasons and an analysis considering the influence of seasonality may be required in future investigations to fully clarify ET community responses to elevation in Lamington National Park.

### 4.3 Indicator Species for Elevational Zones

ET assemblage composition varied across elevational zones. Some taxa were distributed widely across the elevational gradient whilst others had limited elevational distributions. For example, the ephemeropteran *Nousia* sp. A and the trichopterans *Tasimia* sp., *Diplectrona* sp. AV3, and *Chimarra* sp. occurred across the entire 300–1100 m gradient. The genera *Chimarra* sp. (Trichoptera: Philopotamidae) and *Nousia* sp. (Ephemeroptera: Leptophlebiidae) are not endemic to Australia and are widely distributed, with the latter also occurring throughout temperate South America (Pescador and Peters 1985; Finlay 1999). Other taxa widely distributed along the gradient (300–900 m) included the ephemeropterans *Austrophlebiodes* sp. A, Caenid A sp., and Baetid Genus 2 sp., and the trichopteran Genus Con B sp. AV2.

In contrast, many taxa were associated with specific, narrower, elevational ranges. Between the Ephemeroptera and Trichoptera, elevation specific ‘indicator’ species could be successfully identified. The ephemeropterans displayed strong species level elevational fidelity; *Kirrara procera* (300–500 m), *Atalophlebia* sp. AV21 (700–900 m) and *Koorrnonga* sp. (900–1100 m) were all associated with specific elevational zones. *Kirrara procera* and *Koorrnonga* sp. showed strong specificity and fidelity, only occurring within the 300–500 m and 900–1100 m zones, respectively. *Koorrnonga* sp. is a predominantly temperate genus found throughout south‐eastern Australia (Finlay 1999), and this may explain why it was restricted to the highest elevations within Lamington National Park, confined to the edge of upland subtropical rainforest and upland temperate rainforest. In comparison, elevation specificity was weaker for the trichopterans at higher elevations, with stronger associations occurring at lower elevations. The taxa *Helocabbus* sp., *Cheumatopsyche* sp. and *Diplectrona* sp. AV3 were associated with the 300 m, 300–500 m and 500–1100 m elevational zones, respectively.

Due to the strong elevational signal of several taxa and the strong correlation between ET assemblages and water temperature, it is likely that ET taxa could serve as early indicators of climate‐induced shifts in subtropical rainforest streams. Assemblage level responses of insects to climate change have already been observed in both terrestrial (Wilson et al. 2007) and aquatic systems (Baranov et al. 2020) internationally. In the case of Baranov et al. (2020), their results suggest that even in a protected, well‐forested system with little to no anthropogenic influences, climate change has already drastically shifted the composition of stream macroinvertebrate assemblages over the past several decades. Although our results suggest that ET show a strong elevational signal, only one morphospecies was identified as being restricted above 900 m a.s.l. Overall, this would suggest that ET taxa are not of immediate conservation concern due to climate change in Lamington National Park.

### 4.4 Environmental Filters and Metacommunity Dynamics Structuring Assemblages

Contrary to the terrestrial elevational studies conducted in Lamington National Park under the IBISCA model (see Burwell and Nakamura 2011; Lambkin et al. 2011; Boulter et al. 2011), this study involved in situ measurements of habitat and environmental variables at the site scale at the time of sampling. This allowed us to identify how environmental variables change across the gradient and how ET assemblages vary with those changes. Water temperature, stream width, and canopy cover were the three variables that consistently explained the most variation among assemblages; however, these factors also vary predictably across the elevation gradient with generally smaller stream width, lower temperatures, and higher canopy cover at higher elevations (Malcolm et al. 2008; Roth et al. 2010; Garner et al. 2014).

Searching for relationships between diversity and elevation is unlikely to be simplistic as there are important stochastic (e.g., via species dispersal) and deterministic (e.g., abiotic and biotic filtering) processes working to structure aquatic communities through space and time that need to be considered (Hilborn and Stearns 1982; Poff 1997; Logue et al. 2011). For a species to ‘join’ an assemblage at the site scale from a regional species pool in stream systems, it must possess appropriate species traits to ‘pass’ through nested environmental filters (Poff 1997; Graham et al. 2014; Laiolo et al. 2018). Therefore, both ecological and evolutionary explanations are necessary to gain a holistic understanding of elevational diversity gradients (Laiolo et al. 2018; Chiu et al. 2020).

The consideration of broader metacommunity dynamics, species dispersal, and biotic interactions, as well as local environmental factors, physicochemical properties, and microhabitat, can increase the accuracy with which species richness patterns are modelled across elevational gradients in streams (Chiu et al. 2020). The lack of complex modelling techniques and consideration of broader community interactions through a spatiotemporal context could potentially explain why we did not see a clear pattern of species richness for the ephemeropterans in this study and may have provided us with an overly simplistic explanation for the relationship between trichopteran species richness and elevation. Unimodal patterns of species richness across elevational gradients have been widely observed in studies that modelled community composition with metacommunity dynamics and local environmental factors (Bertuzzo et al. 2016; Chiu et al. 2020), whereas monotonic (i.e., consistent) patterns of species richness are often associated with studies that offer a more simplistic perspective on elevation and environmental gradients (Jacobsen et al. 1997; Jung et al. 2008; Henriques‐Oliveira and Nessimian 2010).

ET assemblages at the microhabitat level were strongly grouped by both elevation and microhabitat type. Interestingly, in multivariate space the assemblages at lower elevations (300–500 m) were more similar across catchments than assemblages in the headwater reaches (700–900 m), which were more separated in multidimensional space; this is despite the geographical distance between assemblages at low elevations being greater than between those at high elevations. These atypical patterns of distribution across the gradient are most likely the result of a combination of complex interactions exhibited upon ET populations throughout their life‐cycle, including oviposition and egg fecundity (Reich and Downes 2004; Lancaster et al. 2010; Bovill et al. 2019), advection dispersal (invertebrate drift: Bond et al. 2001; Barquin and Death 2011), and flight dispersal by adults (Hughes et al. 2011; Elizabeth Graham et al. 2017).

## 5 Conclusion

Broadly, this study aimed to characterise Ephemeroptera and Trichoptera assemblages across an elevational gradient in Australian subtropical rainforest streams. Notably, this study was able to demonstrate a clear pattern of environmental change across the elevational gradient of the waterways sampled, and that variations in ET assemblages reflected these environmental changes. Elevation played a significant role in structuring ET assemblages, as did microhabitat type. Variations in water temperature, canopy cover, substrate composition and stream width were most significantly associated with elevation across the gradient, and changes in ET assemblages were strongly associated with these environmental factors. The results of this study suggest that ET assemblages are split between ‘low’ (300 m–500 m a.s.l.) and ‘high’ (700 m–900 m a.s.l.) elevation assemblages within the streams of Lamington National Park. Importantly, several indicator species associated with different elevational zones were identified across the gradient and individual ET morphospecies showed strong elevational signals. However, due to only one morphospecies being restricted to above 900 m a.s.l., it is unlikely that ET taxa are of immediate conservation concern in Lamington National Park. Additionally, this study has provided baseline distributional data on the elevational structure of ET taxa in Lamington National Park that will allow for the monitoring of assemblage level responses to future changes in climate. These data have purpose and value beyond the fundamental establishment of baselines; this study has added a novel and comprehensive insight into the elevational distribution of two orders of insects for which very little information is currently available in Australia.

## Author Contributions

**D. Pagotto:** conceptualization (lead), data curation (lead), formal analysis (lead), investigation (lead), methodology (supporting), writing – original draft (lead), writing – review and editing (supporting). **C. Burwell:** conceptualization (supporting), data curation (supporting), investigation (supporting), methodology (supporting), writing – review and editing (supporting). **K. Turlington:** formal analysis (supporting), writing – review and editing (supporting). **F. Sheldon:** conceptualization (supporting), formal analysis (supporting), investigation (supporting), methodology (supporting), supervision (lead), writing – original draft (supporting), writing – review and editing (supporting).

## Ethics Statement

No permits were required as no live material was collected. All appropriate ethics and other site access approvals were obtained for the research.

## Conflicts of Interest

The authors declare no conflicts of interest.

## Supporting information


**Data S1:** ece373003‐sup‐0001‐DataS1.docx.

**Data S2:** ece373003‐sup‐0002‐DataS2.docx.

## Data Availability

All data not reported in the manuscript are available in the Supporting Information.

## References

[ece373003-bib-0001] Acosta, R. , and N. Prat . 2010. “Chironomid Assemblages in High Altitude Streams of the Andean Region of Peru.” Fundamental and Applied Limnology 177: 57–79. 10.1127/1863-9135/2010/0177-0057.

[ece373003-bib-0002] Alba‐Tercedor, J. , M. Sáinz‐Bariáin , J. M. Poquet , and R. Rodríguez‐López . 2017. “Predicting River Macroinvertebrate Communities Distributional Shifts Under Future Global Change Scenarios in the Spanish Mediterranean Area.” PLoS One 12: e0167904. 28135280 10.1371/journal.pone.0167904 PMC5279736

[ece373003-bib-0003] Ashton, L. A. , R. L. Kitching , S. Maunsell , D. Bito , and D. Putland . 2011. “Macrolepidopteran Assemblages Along an Elevational Gradient in Subtropical Rainforest‐Exploring Indicators of Climate Change.” Memoirs of the Queensland Museum 55: 375–389.

[ece373003-bib-0004] Baggiano, O. , D. J. Schmidt , F. Sheldon , and J. M. Hughes . 2011. “The Role of Altitude and Associated Habitat Stability in Determining Patterns of Population Genetic Structure in Two Species of *Atalophlebia* (Ephemeroptera: Leptophlebiidae).” Freshwater Biology 56: 230–249. 10.1111/j.1365-2427.2010.02490.x.

[ece373003-bib-0005] Baptista, D. , D. Buss , L. Dorville , and J. Nessimian . 2001. “Diversity and Habitat Preference of Aquatic Insects Along the Longitudinal Gradient of the Macaé River Basin, Rio de Janeiro, Brazil.” Revista Brasileira de Biologia 61: 249–258. 10.1590/s0034-71082001000200007. 11514892

[ece373003-bib-0006] Baranov, V. , J. Jourdan , F. Pilotto , R. Wagner , and P. Haase . 2020. “Complex and Nonlinear Climate‐Driven Changes in Freshwater Insect Communities Over 42 Years.” Conservation Biology 34: 1241–1251. 10.1111/cobi.13477. 32022305

[ece373003-bib-0007] Barquin, J. , and R. G. Death . 2011. “Downstream Changes in Spring‐Fed Stream Invertebrate Communities: The Effect of Increased Temperature Range?” Journal of Limnology 70: 134.

[ece373003-bib-0008] Benhadji, N. , S. B. Kurniawan , and M. F. Imron . 2025. “Review of Mayflies (Insecta Ephemeroptera) as a Bioindicator of Heavy Metals and Microplastics in Freshwater.” Science of the Total Environment 958: 178057. 10.1016/j.scitotenv.2024.178057. 39674161

[ece373003-bib-0009] Bertuzzo, E. , F. Carrara , L. Mari , F. Altermatt , I. Rodriguez‐Iturbe , and A. Rinaldo . 2016. “Geomorphic Controls on Elevational Gradients of Species Richness.” Proceedings of the National Academy of Sciences of the United States of America 113: 1737–1742. 26831107 10.1073/pnas.1518922113 PMC4763792

[ece373003-bib-0010] Bond, N. R. , G. L. Perry , and B. J. Downes . 2001. “Dispersal of Organisms in a Patchy Stream Environment Under Different Settlement Scenarios.” Journal of Animal Ecology 69: 608–619.

[ece373003-bib-0011] Boulter, S. L. , C. L. Lambkin , and N. T. Starick . 2011. “Assessing the Abundance of Seven Major Arthropod Groups Along an Altitudinal Gradient and Across Seasons in Subtropical Rainforest.” Memoirs of the Queensland Museum 55: 303–313.

[ece373003-bib-0012] Bovill, W. D. , B. J. Downes , and J. Lancaster . 2019. “Variations in Fecundity Over Catchment Scales: Implications for Caddisfly Populations Spanning a Thermal Gradient.” Freshwater Biology 64: 723–734. 10.1111/fwb.13257.

[ece373003-bib-0013] Bunn, S. E. , E. G. Abal , M. J. Smith , et al. 2010. “Integration of Science and Monitoring of River Ecosystem Health to Guide Investments in Catchment Protection and Rehabilitation.” Freshwater Biology 55, no. S1: 223–240. 10.1111/j.1365-2427.2009.02375.x.

[ece373003-bib-0014] Bureau of Meteorology . 2021. “Climate Change in Australia: Regional Clusters.” https://www.climatechangeinaustralia.gov.au/en/projections‐tools/regional‐climate‐change‐explorer/clusters/?current=ECCandpopup=trueandtooltip=true.

[ece373003-bib-0015] Bureau of Meteorology . 2022. Climate Data Online. Australian Government Retrieved Novermber 2022. https://www.bom.gov.au/climate/.

[ece373003-bib-0016] Burwell, C. J. , and A. Nakamura . 2011. “Distribution of Ant Species Along an Elevational Transect in Continuous Rainforest in Subtropical Queensland, Australia.” Memoirs of the Queensland Museum 55: 391–411.

[ece373003-bib-0017] Bush, A. , V. Hermoso , S. Linke , D. Nipperess , E. Turak , and L. Hughes . 2014. “Freshwater Conservation Planning Under Climate Change: Demonstrating Proactive Approaches for Australian Odonata.” Journal of Applied Ecology 51: 1273–1281. 10.1111/1365-2664.12295.

[ece373003-bib-0018] Castro, D. M. , M. Callisto , R. R. Solar , D. R. Macedo , and G. W. Fernandes . 2019. “Beta Diversity of Aquatic Invertebrates Increases Along an Altitudinal Gradient in a Neotropical Mountain.” Biotropica 51: 399–411. 10.1111/btp.12660.

[ece373003-bib-0019] Chessman, B. C. 2009. “Climatic Changes and 13‐Year Trends in Stream Macroinvertebrate Assemblages in New South Wales, Australia.” Global Change Biology 15: 2791–2802. 10.1111/j.1365-2486.2008.01840.x.

[ece373003-bib-0020] Chiu, M. C. , S. Ao , F. He , V. H. Resh , and Q. Cai . 2020. “Elevation Shapes Biodiversity Patterns Through Metacommunity‐Structuring Processes.” Science of the Total Environment 743: 140548. 32758813 10.1016/j.scitotenv.2020.140548

[ece373003-bib-0021] Dodds, W. K. , L. Bruckerhoff , D. Batzer , et al. 2019. “The Freshwater Biome Gradient Framework: Predicting Macroscale Properties Based on Latitude, Altitude, and Precipitation.” Ecosphere 10: e02786. 10.1002/ecs2.2786.

[ece373003-bib-0022] Dodds, W. K. , K. Gido , M. R. Whiles , M. D. Daniels , and B. P. Grudzinski . 2015. “The Stream Biome Gradient Concept: Factors Controlling Lotic Systems Across Broad Biogeographic Scales.” Freshwater Science 34: 1–19.

[ece373003-bib-0023] dos Reis Oliveira, P. C. , M. H. Kraak , M. Pena‐Ortiz , H. G. van der Geest , and P. F. Verdonschot . 2020. “Responses of Macroinvertebrate Communities to Land Use Specific Sediment Food and Habitat Characteristics in Lowland Streams.” Science of the Total Environment 703: 135060. 10.1016/j.scitotenv.2019.135060. 31757549

[ece373003-bib-0024] Dufrêne, M. , and P. Legendre . 1997. “Species Assemblages and Indicator Species: The Need for a Flexible Asymmetrical Approach.” Ecological Monographs 67: 345–366. 10.1890/0012-9615(1997)067[0345:SAAIST]2.0.CO;2.

[ece373003-bib-0025] Elizabeth Graham, S. , R. Storey , and B. Smith . 2017. “Dispersal Distances of Aquatic Insects: Upstream Crawling by Benthic EPT Larvae and Flight of Adult Trichoptera Along Valley Floors.” New Zealand Journal of Marine and Freshwater Research 51: 146–164. 10.1080/00288330.2016.1268175.

[ece373003-bib-0026] Finlay, K. J. 1999. “Taxonomic and Life History Notes on Australian Nousia and Koorrnonga (Ephemeroptera: Leptophlebiidae).” In The Other 99%: The Conservation and Biodiversity of Invertebrates, edited by W. Ponder and D. Lumney , 196–198. Royal Zoological Society of New South Wales.

[ece373003-bib-0027] Frauendorf, T. C. , R. A. Mackenzie , R. W. Tingley , A. G. Frazier , M. Riney , and R. W. El‐Sabaawi . 2019. “Evaluating Ecosystem Effects of Climate Change on Tropical Island Streams Using High Spatial and Temporal Resolution Sampling Regimes.” Global Change Biology 25: 1344–1357. 10.1111/gcb.14584. 30712279

[ece373003-bib-0028] Frissell, C. A. , W. J. Liss , C. E. Warren , and M. D. Hurley . 1986. “A Hierarchical Framework for Stream Habitat Classification: Viewing Streams in a Watershed Context.” Environmental Management 10: 199–214. 10.1007/BF01867358.

[ece373003-bib-0029] Garner, G. , I. A. Malcolm , J. P. Sadler , and D. M. Hannah . 2014. “What Causes Cooling Water Temperature Gradients in a Forested Stream Reach?” Hydrology and Earth System Sciences 18: 5361–5376. 10.5194/hess-18-5361-2014.

[ece373003-bib-0030] Godoy, B. S. , L. L. Queiroz , S. Lodi , and L. G. Oliveira . 2017. “Environment and Spatial Influences on Aquatic Insect Communities in Cerrado Streams: The Relative Importance of Conductivity, Altitude, and Conservation Areas.” Neotropical Entomology 46: 151–158. 10.1007/s13744-016-0452-4. 27909952

[ece373003-bib-0031] Graham, C. H. , A. C. Carnaval , C. D. Cadena , et al. 2014. “The Origin and Maintenance of Montane Diversity: Integrating Evolutionary and Ecological Processes.” Ecography 37: 711–719. 10.1111/ecog.00578.

[ece373003-bib-0032] Greenslade, P. , and R. L. Kitching . 2011. “Potential Effects of Climatic Warming on the Distribution of Collembola Along an Elevational Transect in Lamington National Park, Queensland, Australia.” Memoirs of the Queensland Museum 55: 333–347.

[ece373003-bib-0033] Haase, P. , F. Pilotto , F. Li , et al. 2019. “Moderate Warming Over the Past 25 Years Has Already Reorganized Stream Invertebrate Communities.” Science of the Total Environment 658: 1531–1538. 10.1016/j.scitotenv.2018.12.234. 30678011

[ece373003-bib-0034] Hagger, V. , D. Fisher , S. Schmidt , and S. Blomberg . 2013. “Assessing the Vulnerability of an Assemblage of Subtropical Rainforest Vertebrate Species to Climate Change in South‐East Queensland.” Austral Ecology 38: 465–475. 10.1111/j.1442-9993.2012.02437.x.

[ece373003-bib-0035] Hawking, J. H. 2000. Keys to Keys—A Guide to Keys and Zoological Information to Identify Invertebrates From Australian Inland Waters, MDFRC Identification Guide 2. CRCFE Albury.

[ece373003-bib-0036] Henriques‐Oliveira, A. , and J. Nessimian . 2010. “Aquatic Macroinvertebrate Diversity and Composition in Streams Along an Elevational Gradient in Southeastern Brazil.” Biota Neotropica 10: 115–128. 10.1590/s1676-06032010000300012.

[ece373003-bib-0102] Hilborn, R. , and S. C. Stearns . 1982. “On Inference in Ecology and Evolutionary Biology: The Problem of Multiple Causes.” Acta Biotheoretica 31, no. 3: 145–164. 10.1007/bf01857238. 6815944

[ece373003-bib-0037] Hodkinson, I. D. 2005. “Terrestrial Insects Along Elevation Gradients: Species and Community Responses to Altitude.” Biological Reviews 80: 489–513. 10.1017/S1464793105006767. 16094810

[ece373003-bib-0038] Hotaling, S. , D. Finn , J. Joseph Giersch , D. Weisrock , and D. Jacobsen . 2017. “Climate Change and Alpine Stream Biology: Progress, Challenges, and Opportunities for the Future.” Biological Reviews 92: 2024–2045. 10.1111/brv.12319. 28105701

[ece373003-bib-0039] Hughes, J. M. , J. A. Huey , A. J. McLean , and O. Baggiano . 2011. “Aquatic Insects in Eastern Australia: A Window on Ecology and Evolution of Dispersal in Streams.” Insects 2: 447–461. https://www.mdpi.com/2075‐4450/2/4/447. 26467824 10.3390/insects2040447 PMC4553437

[ece373003-bib-0040] Jacobsen, D. 2003. “Altitudinal Changes in Diversity of Macroinvertebrates From Small Streams in the Ecuadorian Andes.” Archiv für Hydrobiologie 158: 145–167. 10.1127/0003-9136/2003/0158-0145.

[ece373003-bib-0041] Jacobsen, D. 2007. “Low Oxygen Pressure as a Driving Factor for the Altitudinal Decline in Taxon Richness of Stream Macroinvertebrates.” Oecologia 154: 795–807. 10.1007/s00442-007-0877-x. 17960424

[ece373003-bib-0042] Jacobsen, D. , and A. Encalada . 1998. “The Macroinvertebrate Fauna of Ecuadorian Highland Streams in the Wet and Dry Season.” Archiv für Hydrobiologie 142: 53–70.

[ece373003-bib-0043] Jacobsen, D. , S. Rostgaard , and J. Vásconez . 2003. “Are Macroinvertebrates in High Altitude Streams Affected by Oxygen Deficiency?” Freshwater Biology 48: 2025–2032. 10.1046/j.1365-2427.2003.01140.x.

[ece373003-bib-0044] Jacobsen, D. , R. Schultz , and A. Encalada . 1997. “Structure and Diversity of Stream Invertebrate Assemblages: The Influence of Temperature With Altitude and Latitude.” Freshwater Biology 38: 247–261. 10.1046/j.1365-2427.1997.00210.x.

[ece373003-bib-0045] Jacobus, L. M. , C. R. Macadam , and M. Sartori . 2019. “Mayflies (Ephemeroptera) and Their Contributions to Ecosystem Services.” Insects 10: 170. 10.3390/insects10060170. 31207933 PMC6628430

[ece373003-bib-0046] Jiang, X.‐M. , J. Xiong , J.‐W. Qiu , J.‐M. Wu , J.‐W. Wang , and Z.‐C. Xie . 2010. “Structure of Macroinvertebrate Communities in Relation to Environmental Variables in a Subtropical Asian River System.” International Review of Hydrobiology 95: 42–57. 10.1002/iroh.200811131.

[ece373003-bib-0047] Jung, S. W. , V. V. Nguyen , Q. H. Nguyen , and Y. J. Bae . 2008. “Aquatic Insect Faunas and Communities of a Mountain Stream in Sapa Highland, Northern Vietnam.” Limnology 9: 219–229. 10.1007/s10201-008-0250-8.

[ece373003-bib-0048] Kitching, R. L. , D. Putland , L. A. Ashton , et al. 2011. “Detecting Biodiversity Changes Along Climatic Gradients: The IBISCA‐Queensland Project.” Memoirs of the Queensland Museum 55: 235–250.

[ece373003-bib-0049] Klinges, D. , and B. Scheffers . 2021. “Microgeography, Not Just Latitude, Drives Climate Overlap on Mountains From Tropical to Polar Ecosystems.” American Naturalist 197: 75–92. 10.1086/711873. 33417520

[ece373003-bib-0050] Kottek, M. , J. Grieser , C. Beck , B. Rudolf , and F. Rubel . 2006. “World Map of the Köppen‐Geiger Climate Classification Updated.” Meteorologische Zeitschrift 15, no. 3: 259–263. 10.1127/0941-2948/2006/0130.

[ece373003-bib-0051] Laidlaw, M. J. , W. J. F. McDonald , R. J. Hunter , D. A. Putland , and R. L. Kitching . 2011. “The Potential Impacts of Climate Change on Australian Subtropical Rainforest.” Australian Journal of Botany 59: 440–449. 10.1071/bt10319.

[ece373003-bib-0052] Laiolo, P. , J. Pato , and J. R. Obeso . 2018. “Ecological and Evolutionary Drivers of the Elevational Gradient of Diversity.” Ecology Letters 21: 1022–1032. 10.1111/ele.12967. 29722129

[ece373003-bib-0053] Lambkin, C. L. , S. L. Boulter , N. T. Starick , et al. 2011. “Altitudinal and Seasonal Variation in the Family‐Level Assemblages of Flies (Diptera) in an Australian Subtropical Rainforest: One Hundred Thousand and Counting!” Memoirs of the Queensland Museum 55: 315–331.

[ece373003-bib-0054] Lancaster, J. , B. J. Downes , and A. Arnold . 2010. “Oviposition Site Selectivity of Some Stream‐Dwelling Caddisflies.” Hydrobiologia 652: 165–178. 10.1007/s10750-010-0328-2.

[ece373003-bib-0055] Laurance, W. F. , B. Dell , S. M. Turton , et al. 2011. “The 10 Australian Ecosystems Most Vulnerable to Tipping Points.” Biological Conservation 144: 1472–1480. 10.1016/j.biocon.2011.01.016.

[ece373003-bib-0101] Li, F. , Y. Kwon , M. Bae , N. Chung , T. Kwon , and Y. Park . 2013. “Potential Impacts of Global Warming on the Diversity and Distribution of Stream Insects in South Korea.” Conservation Biology 28, no. 2: 498–508. 10.1111/cobi.12219. 24372690

[ece373003-bib-0056] Logue, J. B. , N. Mouquet , H. Peter , and H. Hillebrand . 2011. “Empirical Approaches to Metacommunities: A Review and Comparison With Theory.” Trends in Ecology & Evolution 26: 482–491. 10.1016/j.tree.2011.04.009. 21641673

[ece373003-bib-0057] Malcolm, I. A. , C. Soulsby , D. M. Hannah , P. J. Bacon , A. F. Youngson , and D. Tetzlaff . 2008. “The Influence of Riparian Woodland on Stream Temperatures: Implications for the Performance of Juvenile Salmonids.” Hydrological Processes 22: 968–979. 10.1002/hyp.6996.

[ece373003-bib-0058] Marshall, J. C. , F. Sheldon , M. Thoms , and S. Choy . 2006. “The Macroinvertebrate Fauna of an Australian Dryland River: Spatial and Temporal Patterns and Environmental Relationships.” Marine and Freshwater Research 57: 61–74. 10.1071/mf05021.

[ece373003-bib-0059] Mazzucco, R. , T. Van Nguyen , D. Kim , T. Chon , and U. Dieckmann . 2015. “Adaptation of Aquatic Insects to the Current Flow in Streams.” Ecological Modelling 309–310: 143–152. 10.1016/j.ecolmodel.2015.04.019.

[ece373003-bib-0060] Md Rawi, C. S. , S. A. Al‐Shami , M. R. Madrus , and A. H. Ahmad . 2014. “Biological and Ecological Diversity of Aquatic Macroinvertebrates in Response to Hydrological and Physicochemical Parameters in Tropical Forest Streams of Gunung Tebu, Malaysia: Implications for Ecohydrological Assessment.” Ecohydrology 7: 496–507. 10.1002/eco.1368.

[ece373003-bib-0061] Meadows, P. S. , and J. I. Campbell . 1972. “Habitat Selection by Aquatic Invertebrates.” In Advances in Marine Biology, edited by F. S. Russell and M. Yonge , vol. 10, 271–382. Academic Press. 10.1016/S0065-2881(08)60418-6.

[ece373003-bib-0062] Miserendino, M. L. 2001. “Macroinvertebrate Assemblages in Andean Patagonian Rivers and Streams: Environmental Relationships.” Hydrobiologia 444: 147–158. 10.1023/A:1017519216789.

[ece373003-bib-0063] Monk, W. A. , P. J. Wood , D. M. Hannah , D. A. Wilson , C. A. Extence , and R. P. Chadd . 2006. “Flow Variability and Macroinvertebrate Community Response Within Riverine Systems.” River Research and Applications 22: 595–615. 10.1002/rra.933.

[ece373003-bib-0064] Múrria, C. , A. Rugenski , M. Whiles , and A. Vogler . 2015. “Long‐Term Isolation and Endemicity of Neotropical Aquatic Insects Limit the Community Responses to Recent Amphibian Decline.” Diversity and Distributions 21: 938–949. 10.1111/ddi.12343.

[ece373003-bib-0065] Nakamura, A. , C. J. Burwell , L. A. Ashton , M. J. Laidlaw , M. Katabuchi , and R. L. Kitching . 2016. “Identifying Indicator Species of Elevation: Comparing the Utility of Woody Plants, Ants and Moths for Long‐Term Monitoring.” Austral Ecology 41: 179–188. 10.1111/aec.1229.

[ece373003-bib-0066] Nguyen, T. H. T. , M. A. E. Forio , P. Boets , et al. 2018. “Threshold Responses of Macroinvertebrate Communities to Stream Velocity in Relation to Hydropower Dam: A Case Study From the Guayas River Basin (Ecuador).” Water 10: 1195. 10.3390/w10091195.

[ece373003-bib-0067] Nukazawa, K. , R. Arai , S. Kazama , and Y. Takemon . 2018. “Projection of Invertebrate Populations in the Headwater Streams of a Temperate Catchment Under a Changing Climate.” Science of the Total Environment 642: 610–618. 10.1016/j.scitotenv.2018.06.109. 29909328

[ece373003-bib-0068] Ødegaard, F. , and O. H. Diserud . 2011. “Taxonomic Composition of Coleoptera, Hemiptera (Heteroptera and Coleorrhyncha) and Mutillidae (Hymenoptera) at Five Different Altitudes in Lamington National Park (Queensland, Australia).” Memoirs of the Queensland Museum 55: 359–374.

[ece373003-bib-0069] Park, Y. S. , R. Céréghino , A. Compin , and S. Lek . 2003. “Applications of Artificial Neural Networks for Patterning and Predicting Aquatic Insect Species Richness in Running Waters.” Ecological Modelling 160: 265–280. 10.1016/S0304-3800(02)00258-2.

[ece373003-bib-0070] Pescador, M. L. , and W. L. Peters . 1985. “Biosystematics of the Genus *Nousia* From Southern South America (Ephemeroptera: Leptophlebiidae: Atalophlebiinae).” Journal of the Kansas Entomological Society 58: 91–123.

[ece373003-bib-0071] Poff, N. L. 1997. “Landscape Filters and Species Traits: Towards Mechanistic Understanding and Prediction in Stream Ecology.” Journal of the North American Benthological Society 16: 391–409. 10.2307/1468026.

[ece373003-bib-0072] Principe, R. E. , J. A. Márquez , and L. Cibils‐Martina . 2019. “Distribution and Habitat Preference of Ephemeroptera and Trichoptera in Subtropical Mountain Streams: Implications for Monitoring and Conservation.” Anais da Academia Brasileira de Ciências 91, no. 3: e20180692. 10.1590/0001-3765201920180692. 31618411

[ece373003-bib-0073] Pyne, M. I. , and N. L. Poff . 2017. “Vulnerability of Stream Community Composition and Function to Projected Thermal Warming and Hydrologic Change Across Ecoregions in the Western United States.” Global Change Biology 23: 77–93. 10.1111/gcb.13437. 27429092

[ece373003-bib-0074] Queensland Globe . 2022. “Qldglobe.information.qld.gov.au.” https://qldglobe.information.qld.gov.au/.

[ece373003-bib-0075] Queensland Government Regional Ecosystems Descriptions Database . 2021. “Regional Ecosystem Descriptions.” https://www.qld.gov.au/environment/plants‐animals/plants/ecosystems/descriptions.

[ece373003-bib-0077] Reich, P. , and B. J. Downes . 2004. “Relating Larval Distributions to Patterns of Oviposition: Evidence From Lotic Hydrobiosid Caddisflies.” Freshwater Biology 49: 1423–1436. 10.1111/j.1365-2427.2004.01278.x.

[ece373003-bib-0078] Roth, T. R. , M. C. Westhoff , H. Huwald , et al. 2010. “Stream Temperature Response to Three Riparian Vegetation Scenarios by Use of a Distributed Temperature Validated Model.” Environmental Science & Technology 44: 2072–2078. 10.1021/es902654f. 20131784

[ece373003-bib-0079] Sauer, J. , S. Domisch , C. Nowak , and P. Haase . 2011. “Low Mountain Ranges: Summit Traps for Montane Freshwater Species Under Climate Change.” Biodiversity and Conservation 20: 3133–3146. 10.1007/s10531-011-0140-y.

[ece373003-bib-0080] Schmidt, C. , A. Musolff , N. Trauth , M. Vieweg , and J. Fleckenstein . 2012. “Transient Analysis of Fluctuations of Electrical Conductivity as Tracer in the Stream Bed.” Hydrology and Earth System Sciences 16: 3689–3697. 10.5194/hess-16-3689-2012.

[ece373003-bib-0081] Sheldon, F. , A. J. Boulton , and J. T. Puckridge . 2002. “Conservation Value of Variable Connectivity: Aquatic Invertebrate Assemblages of Channel and Floodplain Habitats of a Central Australian Arid‐Zone River, Cooper Creek.” Biological Conservation 103: 13–31. 10.1016/s0006-3207(01)00111-2.

[ece373003-bib-0082] Sponseller, R. A. , E. F. Benfield , and H. M. Valett . 2001. “Relationships Between Land Use, Spatial Scale and Stream Macroinvertebrate Communities.” Freshwater Biology 46: 1409–1424. 10.1046/j.1365-2427.2001.00758.x.

[ece373003-bib-0083] Strong, C. , S. L. Boulter , M. J. Laidlaw , S. C. Maunsell , D. Putland , and R. L. Kitching . 2011. “The Physical Environment of an Altitudinal Gradient in the Rainforest of Lamington National Park, Southeast Queensland.” Memoirs of the Queensland Museum—Nature 55: 251–270.

[ece373003-bib-0084] Suren, A. M. 1994. “Macroinvertebrate Communities of Streams in Western Nepal: Effects of Altitude and Land Use.” Freshwater Biology 32: 323–336. 10.1111/j.1365-2427.1994.tb01129.x.

[ece373003-bib-0086] Vannote, R. , G. Minshall , K. Cummins , J. Sedell , and C. Cushing . 1980. “The River Continuum Concept.” Canadian Journal of Fisheries and Aquatic Sciences 37: 130–137. 10.1139/f80-017.

[ece373003-bib-0087] Ward, J. V. 1989. “The Four‐Dimensional Nature of Lotic Ecosystems.” Journal of the North American Benthological Society 8: 2–8. 10.2307/1467397.

[ece373003-bib-0088] Ward, J. V. , and J. A. Stanford . 1982. “Thermal Responses in the Evolutionary Ecology of Aquatic Insects.” Annual Review of Entomology 27: 97–117. 10.1146/annurev.en.27.010182.000525.

[ece373003-bib-0089] Waterwatch Australia Steering Committee . 2002. “Module 4—Physical and Chemical Parameters.” http://www.vic.waterwatch.org.au/resources/water_watch_module4_physical_and_chemical.pdf.

[ece373003-bib-0090] Wilson, R. J. , D. Gutierrez , J. Gutierrez , and V. J. Monserrat . 2007. “An Elevational Shift in Butterfly Species Richness and Composition Accompanying Recent Climate Change.” Global Change Biology 13: 1873–1887. 10.1111/j.1365-2486.2007.01418.x.

[ece373003-bib-0091] Yegon, M. , F. Masese , A. Sitati , and W. Graf . 2021. “Elevation and Land Use as Drivers of Macroinvertebrate Functional Composition in Afromontane Headwater Streams.” Marine and Freshwater Research 72: 1517. 10.1071/mf21048.

[ece373003-bib-0092] Young, B. A. , D. J. Schmidt , and F. Sheldon . 2013. “Small‐Scale Patterns of Genetic Variation in a Headwater Specialist Mayfly: No Influence of Selective Forest Harvesting on Diversity.” Austral Ecology 38: 504–515. 10.1111/j.1442-9993.2012.02440.x.

