# Supplementary material for: Ephemeropteran and Trichopteran Assemblages Vary Across a Subtropical Rainforest Altitudinal Gradient: Useful Indicators for Climate Change
Source: Ecol Evol. 2026 Feb 2;16(2):e73003. doi: 10.1002/ece3.73003 (PMC12862282; doi:10.1002/ece3.73003)
Supplement: Supplementary file 1 — Data S1: ece373003‐sup‐0001‐DataS1.docx. [file ECE3-16-e73003-s002.docx]

**Appendix 1**: List of survey sites, including relevant catchment, site abbreviations, elevation, and number of microhabitats sampled. m.a.s.l = metres above sea level.

| **Catchment** | **Site Code** | **Latitude and Longitude** | **Elevation (m.a.s.l)** | **Microhabitats^1^** | **No. of Samples** |
| --- | --- | --- | --- | --- | --- |
| **Albert River**  **Albert River**  **Albert River**  **Albert River** | **AR1** | -28.26509 153.11638 | 350 | Run  Riffle  Pool | 3  3  3 |
|  | **AR2** | -28.27659 153.14391 | 440 | Run  Riffle  Pool | 3  3  3 |
|  | **AR3** | -28.23212  153.12624 | 775 | Run  Riffle  Pool | 3  3  3 |
|  | **AR4** | -28.24152 153.13827 | 885 | Run  Riffle  Pool | 3  3  3 |
| **Canungra Creek**  **Canungra Creek**  **Canungra Creek**  **Canungra Creek**  **Canungra Creek** | **CC1** | -28.15796 153.13690 | 265 | Run  Riffle  Pool | 3  3  3 |
|  | **CC2** | -28.21710 153.14071 | 495 | Run  Riffle  Pool | 3  3  2 |
|  | **CC3** | -28.23659 153.15150 | 690 | Run  Riffle  Pool | 3  3  3 |
|  | **CC4** | -28.24503  153.16380 | 850 | Run  Riffle  Pool | 3  3  2 |
|  | **CC5** | -28.25240 153.17243 | 1135 | Run  Riffle  Pool | 3  3  2 |
| **Coomera River**  **Coomera River**  **Coomera River**  **Coomera River** | **CR1** | -28.16814 153.16910 | 305 | Run  Riffle  Pool | 3  3  2 |
|  | **CR2** | -28.21131 153.18127 | 425 | Run  Riffle  Pool | 3  3  3 |
|  | **CR3** | -28.23602  153.19292 | 725 | Run  Riffle  Pool | 3  3  3 |
|  | **CR4** | -28.24887  153.19859 | 885 | Run  Riffle  Pool | 3  3  3 |

**Appendix 2**: List of Ephemeroptera and Trichoptera taxa collected in Lamington National Park; **Site codes: CR =** Coomera River; **CC =** Canungra Creek; **AR =** Albert River. **1** = 300m; **2** = 500m; **3** = 700m; **4** = 900m; **5** = 1100m. An ‘x’ marks the presence of the morphospecies at that site.

| **Species/Morphospecies** | **AR1** | **AR2** | **AR3** | **AR4** | **CC1** | **CC2** | **CC3** | **CC4** | **CC5** | **CR1** | **CR2** | **CR3** | **CR4** |
| --- | --- | --- | --- | --- | --- | --- | --- | --- | --- | --- | --- | --- | --- |
|  | **300m** | **500m** | **700m** | **900m** | **300m** | **500m** | **700m** | **900m** | **1100m** | **300m** | **500m** | **700m** | **900m** |
| **Ephemeroptera** |  |  |  |  |  |  |  |  |  |  |  |  |  |
| **Ameletopsidae** |  |  |  |  |  |  |  |  |  |  |  |  |  |
| *Mirawara* sp. |  |  |  |  | x |  |  |  |  |  | x |  |  |
| **Baetidae** |  |  |  |  |  |  |  |  |  |  |  |  |  |
| Baetid Genus 2 sp. | x | x | x | x | x | x | x | x |  | x | x | x | x |
| *Bungona* sp. |  |  |  |  |  |  |  | x |  |  |  |  |  |
| *Centroptilum* sp. | x |  | x |  | x | x |  |  |  | x | x |  |  |
| **Caenidae** |  |  |  |  |  |  |  |  |  |  |  |  |  |
| Caenid A sp. | x | x | x |  | x | x | x | x |  | x | x | x | x |
| Caenid B sp. |  |  |  |  | x |  |  |  |  |  |  |  |  |
| *Wundacaenis flabellum* |  |  |  |  | x |  |  |  |  | x |  |  |  |
| **Leptophlebiidae** |  |  |  |  |  |  |  |  |  |  |  |  |  |
| *Atalomicria* sp. |  |  |  | x |  |  |  | x |  | x |  |  |  |
| *Atalomicria* sp. AV1 |  |  | x | x |  |  |  |  |  |  |  |  |  |
| *Atalophlebia albiterminata* | x |  |  |  | x |  |  |  |  | x | x |  |  |
| *Atalophlebia* sp. |  |  |  |  | x |  | x |  |  |  |  |  |  |
| *Atalophlebia* sp. AV13 |  |  |  |  | x |  |  |  |  |  |  |  |  |
| *Atalophlebia* sp.AV2 | x |  |  |  |  |  |  |  |  |  |  |  |  |
| *Atalophlebia* sp. AV21 | x |  | x | x |  |  | x | x |  |  |  | x | x |
| *Atalophlebia* sp. AV8 |  |  |  |  |  |  |  |  |  |  |  |  | x |
| *Austrophlebioides* sp. AV10 |  |  |  |  |  | x |  |  |  |  |  |  |  |
| *Austrophlebioides* sp. A | x | x | x | x | x | x | x | x |  | x | x | x | x |
| *Austrophlebioides* sp. AV9 |  |  |  |  |  |  |  | x |  |  |  | x | x |
| Genus O sp. AV1 |  |  |  |  |  |  | x |  |  |  |  | x |  |
| *Jappa* sp. AV2 |  |  |  |  | x |  |  |  |  |  |  |  |  |
| *Jappa* sp. AV3 | x |  |  |  |  |  |  | x |  | x | x |  |  |
| *Kirrara procera* | x | x |  |  | x | x |  |  |  | x | x |  |  |
| *Koorrnonga* sp. |  |  |  | x |  |  |  | x | x |  |  |  | x |
| Leptophlebiidae sp. A |  |  |  | x |  |  |  |  |  | x | x | x | x |
| Leptophlebiidae sp. B |  |  |  | x |  |  |  |  |  | x |  | x |  |
| *Nousia* sp. A |  | x | x | x |  |  | x | x | x | x | x | x | x |
| *Nousia* sp. AV2 | x | x | x | x |  | x | x | x |  | x | x | x | x |
| *Ulmerophlebia* sp. AV2 | x | x | x | x | x | x | x | x |  | x | x | x | x |
| **Vietnamellidae** |  |  |  |  |  |  |  |  |  |  |  |  |  |
| *Austremerella picta* |  | x | x | x |  |  | x |  |  |  |  |  | x |
| **Trichoptera** |  |  |  |  |  |  |  |  |  |  |  |  |  |
| **Antipodoeciidae** |  |  |  |  |  |  |  |  |  |  |  |  |  |
| *Antipodoecia* sp. AV2 | x |  |  |  |  |  | x | x |  |  |  | x | x |
| **Calamoceratidae** |  |  |  |  |  |  |  |  |  |  |  |  |  |
| *Anisocentropus* sp. | x |  | x |  | x | x | x |  |  | x |  | x |  |
| **Calocidae** |  |  |  |  |  |  |  |  |  |  |  |  |  |
| *Caenota plicata* |  |  | x |  |  |  |  |  | x |  |  |  |  |
| *Caloca* sp. |  |  |  |  |  |  | x |  |  |  |  |  | x |
| Genus Cal D sp. AV2 | x | x |  | x |  | x | x | x |  | x | x |  |  |
| *Pliocaloca* sp. AV1 | x |  |  |  |  |  |  |  |  |  |  |  |  |
| *Tamasia variegata* | x |  |  |  |  | x |  |  |  | x | x |  |  |
| **Conoesucidae** |  |  |  |  |  |  |  |  |  |  |  |  |  |
| *Coenoria* sp. |  |  |  |  | x |  |  |  |  | x |  |  |  |
| *Conoesucus* sp. AV4 |  |  |  |  |  |  | x | x |  |  |  |  |  |
| *Costora* sp. AV2 |  |  |  |  |  |  |  | x |  |  |  |  |  |
| Genus Con B sp. AV2 |  | x |  |  | x | x | x | x |  |  | x | x |  |
| **Ecnomidae** |  |  |  |  |  |  |  |  |  |  |  |  |  |
| *Daternomina* sp. | x |  | x |  | x | x |  |  |  | x |  |  |  |
| Ecnomidae Genus D sp. |  |  |  | x |  |  | x |  |  |  |  |  |  |
| *Ecnomina* sp. |  | x |  |  |  |  | x |  |  |  |  |  | x |
| *Ecnomus* sp. | x | x | x |  |  |  |  |  |  |  |  |  |  |
| **Glossosomatidae** |  |  |  |  |  |  |  |  |  |  |  |  |  |
| *Agapetus* sp. AV1 | x | x | x |  | x | x |  | x |  | x | x | x | x |
| **Helicophidae** |  |  |  |  |  |  |  |  |  |  |  |  |  |
| Genus Cal C sp. AV2 |  |  |  |  |  |  |  | x |  |  |  |  |  |
| Genus Hel C sp. AV2 |  |  |  |  |  |  | x |  | x |  |  |  |  |
| *Heloccabus* sp. | x |  |  |  | x | x |  |  |  | x | x |  |  |
| **Helicopsychidae** |  |  |  |  |  |  |  |  |  |  |  |  |  |
| *Helicopsyche* sp. | x |  |  |  |  |  |  |  |  |  | x |  |  |
| **Hydrobiosidae** |  |  |  |  |  |  |  |  |  |  |  |  |  |
| *Apsilochorema gisbum* | x |  | x |  | x |  | x |  |  | x |  | x | x |
| *Ethochorema brunneum* | x |  | x |  | x | x | x |  |  | x |  |  |  |
| Genus sp. A |  |  |  |  |  |  |  | x |  |  |  |  |  |
| *Psyllobetina cumberlandica* |  |  | x |  |  |  |  |  |  |  |  |  |  |
| *Psyllobetina locula* |  |  | x |  |  |  |  |  |  |  |  |  |  |
| **Hydropsychidae** |  |  |  |  |  |  |  |  |  |  |  |  |  |
| *Asmicridea* sp. AV1 |  |  | x |  | x | x |  |  |  | x | x |  | x |
| *Baliomorpha* sp. |  |  |  |  | x |  |  |  |  |  |  |  |  |
| *Cheumatopsyche* sp. A | x | x |  |  | x | x |  |  |  | x | x |  |  |
| *Diplectrona* sp. AV1 |  |  |  | x |  |  |  |  | x |  |  | x | x |
| *Diplectrona* sp. AV11 |  |  | x | x |  |  |  |  |  |  |  |  |  |
| *Diplectrona* sp. AV3 | x | x | x | x |  | x | x | x | x |  | x | x | x |
| **Leptoceridae** |  |  |  |  |  |  |  |  |  |  |  |  |  |
| *Notalina* sp. | x | x | x |  |  |  | x | x |  |  | x |  |  |
| *Triplectides australicus* |  | x |  |  | x |  |  |  |  | x |  |  |  |
| *Triplectides* sp. AV10 | x |  |  |  | x | x | x |  |  |  | x |  |  |
| **Odontoceridae** |  |  |  |  |  |  |  |  |  |  |  |  |  |
| *Barynema costatum* | x |  |  |  |  | x | x |  |  |  |  | x | x |
| *Marilia bola* |  | x |  |  | x |  |  |  |  | x | x |  |  |
| **Philopotamidae** |  |  |  |  |  |  |  |  |  |  |  |  |  |
| *Chimarra* sp. |  |  |  |  | x | x |  |  | x |  |  |  |  |
| *Hydrobiosella* sp. | x |  |  | x |  | x | x |  |  |  | x |  | x |
| **Philorheithridae** |  |  |  |  |  |  |  |  |  |  |  |  |  |
| Genus Philor A sp. A | x |  |  |  |  |  |  |  |  | x |  |  |  |
| *Kosrheithrus* sp. |  |  |  |  | x |  |  |  |  |  |  |  |  |
| *Philorheithrus* sp. |  |  |  |  |  | x |  |  |  |  |  |  |  |
| **Polycentropodidae** |  |  |  |  |  |  |  |  |  |  |  |  |  |
| Genus I sp. AV2 |  |  | x |  |  | x |  | x |  |  |  |  |  |
| *Paranyctiophylax* sp. AV5 |  |  | x |  | x |  |  |  |  |  |  |  |  |
| *Plectrocnemia* sp. AV1 | x |  |  |  |  |  |  |  |  |  |  |  |  |
| **Tasimiidae** |  |  |  |  |  |  |  |  |  |  |  |  |  |
| *Tasimia* sp. | x | x | x | x |  | x | x | x | x |  | x | x | x |
