# Supplementary material for: Ephemeropteran and Trichopteran Assemblages Vary Across a Subtropical Rainforest Altitudinal Gradient: Useful Indicators for Climate Change
Source: Ecol Evol. 2026 Feb 2;16(2):e73003. doi: 10.1002/ece3.73003 (PMC12862282; doi:10.1002/ece3.73003)
Supplement: Supplementary file 2 — Data S2: ece373003‐sup‐0002‐DataS2.docx. [file ECE3-16-e73003-s001.docx]

**Supplemental Information for:**

**Ephemeropteran and Trichopteran assemblages vary across a sub-tropical rainforest altitudinal gradient: useful indicators for climate change.**

Pagotto, D^2^., Burwell, C.^2,3^ and Sheldon, F.^1,2*^

^1^*Australian Rivers Institute, Griffith University, QLD, Australia*

^2^*School of Environment and Science, Griffith University, QLD, Australia*

^3^*Queensland Museum, Brisbane, QLD, Australia*

List of Ephemeroptera and Trichoptera taxa collected in Lamington National Park; **Site codes: CR =** Coomera River; **CC =** Canungra Creek; **AR =** Albert River. **1** = 300m; **2** = 500m; **3** = 700m; **4** = 900m; **5** = 1100m. An ‘x’ marks the presence of the morphospecies at that site.

| **Species/Morphospecies** | **AR1** | **AR2** | **AR3** | **AR4** | **CC1** | **CC2** | **CC3** | **CC4** | **CC5** | **CR1** | **CR2** | **CR3** | **CR4** |
| --- | --- | --- | --- | --- | --- | --- | --- | --- | --- | --- | --- | --- | --- |
|  | **300m** | **500m** | **700m** | **900m** | **300m** | **500m** | **700m** | **900m** | **1100m** | **300m** | **500m** | **700m** | **900m** |
| **Ephemeroptera** |  |  |  |  |  |  |  |  |  |  |  |  |  |
| **Ameletopsidae** |  |  |  |  |  |  |  |  |  |  |  |  |  |
| *Mirawara* sp. |  |  |  |  | x |  |  |  |  |  | x |  |  |
| **Baetidae** |  |  |  |  |  |  |  |  |  |  |  |  |  |
| Baetid Genus 2 sp. | x | x | x | x | x | x | x | x |  | x | x | x | x |
| *Bungona* sp. |  |  |  |  |  |  |  | x |  |  |  |  |  |
| *Centroptilum* sp. | x |  | x |  | x | x |  |  |  | x | x |  |  |
| **Caenidae** |  |  |  |  |  |  |  |  |  |  |  |  |  |
| Caenid A sp. | x | x | x |  | x | x | x | x |  | x | x | x | x |
| Caenid B sp. |  |  |  |  | x |  |  |  |  |  |  |  |  |
| *Wundacaenis flabellum* |  |  |  |  | x |  |  |  |  | x |  |  |  |
| **Leptophlebiidae** |  |  |  |  |  |  |  |  |  |  |  |  |  |
| *Atalomicria* sp. |  |  |  | x |  |  |  | x |  | x |  |  |  |
| *Atalomicria* sp. AV1 |  |  | x | x |  |  |  |  |  |  |  |  |  |
| *Atalophlebia albiterminata* | x |  |  |  | x |  |  |  |  | x | x |  |  |
| *Atalophlebia* sp. |  |  |  |  | x |  | x |  |  |  |  |  |  |
| *Atalophlebia* sp. AV13 |  |  |  |  | x |  |  |  |  |  |  |  |  |
| *Atalophlebia* sp.AV2 | x |  |  |  |  |  |  |  |  |  |  |  |  |
| *Atalophlebia* sp. AV21 | x |  | x | x |  |  | x | x |  |  |  | x | x |
| *Atalophlebia* sp. AV8 |  |  |  |  |  |  |  |  |  |  |  |  | x |
| *Austrophlebioides* sp. AV10 |  |  |  |  |  | x |  |  |  |  |  |  |  |
| *Austrophlebioides* sp. A | x | x | x | x | x | x | x | x |  | x | x | x | x |
| *Austrophlebioides* sp. AV9 |  |  |  |  |  |  |  | x |  |  |  | x | x |
| Genus O sp. AV1 |  |  |  |  |  |  | x |  |  |  |  | x |  |
| *Jappa* sp. AV2 |  |  |  |  | x |  |  |  |  |  |  |  |  |
| *Jappa* sp. AV3 | x |  |  |  |  |  |  | x |  | x | x |  |  |
| *Kirrara procera* | x | x |  |  | x | x |  |  |  | x | x |  |  |
| *Koorrnonga* sp. |  |  |  | x |  |  |  | x | x |  |  |  | x |
| Leptophlebiidae sp. A |  |  |  | x |  |  |  |  |  | x | x | x | x |
| Leptophlebiidae sp. B |  |  |  | x |  |  |  |  |  | x |  | x |  |
| *Nousia* sp. A |  | x | x | x |  |  | x | x | x | x | x | x | x |
| *Nousia* sp. AV2 | x | x | x | x |  | x | x | x |  | x | x | x | x |
| *Ulmerophlebia* sp. AV2 | x | x | x | x | x | x | x | x |  | x | x | x | x |
| **Vietnamellidae** |  |  |  |  |  |  |  |  |  |  |  |  |  |
| *Austremerella picta* |  | x | x | x |  |  | x |  |  |  |  |  | x |
| **Trichoptera** |  |  |  |  |  |  |  |  |  |  |  |  |  |
| **Antipodoeciidae** |  |  |  |  |  |  |  |  |  |  |  |  |  |
| *Antipodoecia* sp. AV2 | x |  |  |  |  |  | x | x |  |  |  | x | x |
| **Calamoceratidae** |  |  |  |  |  |  |  |  |  |  |  |  |  |
| *Anisocentropus* sp. | x |  | x |  | x | x | x |  |  | x |  | x |  |
| **Calocidae** |  |  |  |  |  |  |  |  |  |  |  |  |  |
| *Caenota plicata* |  |  | x |  |  |  |  |  | x |  |  |  |  |
| *Caloca* sp. |  |  |  |  |  |  | x |  |  |  |  |  | x |
| Genus Cal D sp. AV2 | x | x |  | x |  | x | x | x |  | x | x |  |  |
| *Pliocaloca* sp. AV1 | x |  |  |  |  |  |  |  |  |  |  |  |  |
| *Tamasia variegata* | x |  |  |  |  | x |  |  |  | x | x |  |  |
| **Conoesucidae** |  |  |  |  |  |  |  |  |  |  |  |  |  |
| *Coenoria* sp. |  |  |  |  | x |  |  |  |  | x |  |  |  |
| *Conoesucus* sp. AV4 |  |  |  |  |  |  | x | x |  |  |  |  |  |
| *Costora* sp. AV2 |  |  |  |  |  |  |  | x |  |  |  |  |  |
| Genus Con B sp. AV2 |  | x |  |  | x | x | x | x |  |  | x | x |  |
| **Ecnomidae** |  |  |  |  |  |  |  |  |  |  |  |  |  |
| *Daternomina* sp. | x |  | x |  | x | x |  |  |  | x |  |  |  |
| Ecnomidae Genus D sp. |  |  |  | x |  |  | x |  |  |  |  |  |  |
| *Ecnomina* sp. |  | x |  |  |  |  | x |  |  |  |  |  | x |
| *Ecnomus* sp. | x | x | x |  |  |  |  |  |  |  |  |  |  |
| **Glossosomatidae** |  |  |  |  |  |  |  |  |  |  |  |  |  |
| *Agapetus* sp. AV1 | x | x | x |  | x | x |  | x |  | x | x | x | x |
| **Helicophidae** |  |  |  |  |  |  |  |  |  |  |  |  |  |
| Genus Cal C sp. AV2 |  |  |  |  |  |  |  | x |  |  |  |  |  |
| Genus Hel C sp. AV2 |  |  |  |  |  |  | x |  | x |  |  |  |  |
| *Heloccabus* sp. | x |  |  |  | x | x |  |  |  | x | x |  |  |
| **Helicopsychidae** |  |  |  |  |  |  |  |  |  |  |  |  |  |
| *Helicopsyche* sp. | x |  |  |  |  |  |  |  |  |  | x |  |  |
| **Hydrobiosidae** |  |  |  |  |  |  |  |  |  |  |  |  |  |
| *Apsilochorema gisbum* | x |  | x |  | x |  | x |  |  | x |  | x | x |
| *Ethochorema brunneum* | x |  | x |  | x | x | x |  |  | x |  |  |  |
| Genus sp. A |  |  |  |  |  |  |  | x |  |  |  |  |  |
| *Psyllobetina cumberlandica* |  |  | x |  |  |  |  |  |  |  |  |  |  |
| *Psyllobetina locula* |  |  | x |  |  |  |  |  |  |  |  |  |  |
| **Hydropsychidae** |  |  |  |  |  |  |  |  |  |  |  |  |  |
| *Asmicridea* sp. AV1 |  |  | x |  | x | x |  |  |  | x | x |  | x |
| *Baliomorpha* sp. |  |  |  |  | x |  |  |  |  |  |  |  |  |
| *Cheumatopsyche* sp. A | x | x |  |  | x | x |  |  |  | x | x |  |  |
| *Diplectrona* sp. AV1 |  |  |  | x |  |  |  |  | x |  |  | x | x |
| *Diplectrona* sp. AV11 |  |  | x | x |  |  |  |  |  |  |  |  |  |
| *Diplectrona* sp. AV3 | x | x | x | x |  | x | x | x | x |  | x | x | x |
| **Leptoceridae** |  |  |  |  |  |  |  |  |  |  |  |  |  |
| *Notalina* sp. | x | x | x |  |  |  | x | x |  |  | x |  |  |
| *Triplectides australicus* |  | x |  |  | x |  |  |  |  | x |  |  |  |
| *Triplectides* sp. AV10 | x |  |  |  | x | x | x |  |  |  | x |  |  |
| **Odontoceridae** |  |  |  |  |  |  |  |  |  |  |  |  |  |
| *Barynema costatum* | x |  |  |  |  | x | x |  |  |  |  | x | x |
| *Marilia bola* |  | x |  |  | x |  |  |  |  | x | x |  |  |
| **Philopotamidae** |  |  |  |  |  |  |  |  |  |  |  |  |  |
| *Chimarra* sp. |  |  |  |  | x | x |  |  | x |  |  |  |  |
| *Hydrobiosella* sp. | x |  |  | x |  | x | x |  |  |  | x |  | x |
| **Philorheithridae** |  |  |  |  |  |  |  |  |  |  |  |  |  |
| Genus Philor A sp. A | x |  |  |  |  |  |  |  |  | x |  |  |  |
| *Kosrheithrus* sp. |  |  |  |  | x |  |  |  |  |  |  |  |  |
| *Philorheithrus* sp. |  |  |  |  |  | x |  |  |  |  |  |  |  |
| **Polycentropodidae** |  |  |  |  |  |  |  |  |  |  |  |  |  |
| Genus I sp. AV2 |  |  | x |  |  | x |  | x |  |  |  |  |  |
| *Paranyctiophylax* sp. AV5 |  |  | x |  | x |  |  |  |  |  |  |  |  |
| *Plectrocnemia* sp. AV1 | x |  |  |  |  |  |  |  |  |  |  |  |  |
| **Tasimiidae** |  |  |  |  |  |  |  |  |  |  |  |  |  |
| *Tasimia* sp. | x | x | x | x |  | x | x | x | x |  | x | x | x |
